# Aminoribosylated Analogues of Muraymycin Nucleoside Antibiotics

**DOI:** 10.3390/molecules23123085

**Published:** 2018-11-26

**Authors:** Daniel Wiegmann, Stefan Koppermann, Christian Ducho

**Affiliations:** Department of Pharmacy, Pharmaceutical and Medicinal Chemistry, Saarland University, Campus C2 3, 66123 Saarbrücken, Germany; wiegmann_d@web.de (D.W.); stefan.koppermann@uni-saarland.de (S.K.)

**Keywords:** antibiotics, natural products, nucleoside analogues, structure-activity relationships

## Abstract

Nucleoside antibiotics are uridine-derived natural products that inhibit the bacterial membrane protein MraY. MraY is a key enzyme in the membrane-associated intracellular stages of peptidoglycan biosynthesis and therefore considered to be a promising, yet unexploited target for novel antibacterial agents. Muraymycins are one subclass of such naturally occurring MraY inhibitors. As part of structure-activity relationship (SAR) studies on muraymycins and their analogues, we now report on novel derivatives with different attachment of one characteristic structural motif, i.e., the aminoribose moiety normally linked to the muraymycin glycyluridine core unit. Based on considerations derived from an X-ray co-crystal structure, we designed and synthesised muraymycin analogues having the aminoribose attached (via a linker) to either the glycyluridine amino group or to the uracil nucleobase. Reference compounds bearing the non-aminoribosylated linker units were also prepared. It was found that the novel aminoribosylated analogues were inactive as MraY inhibitors in vitro, but that the glycyluridine-modified reference compound retained most of the inhibitory potency relative to the unmodified parent muraymycin analogue. These results point to 6′*-N-*alkylated muraymycin analogues as a potential novel variation of the muraymycin scaffold for future SAR optimisation.

## 1. Introduction

**Scheme 1:**
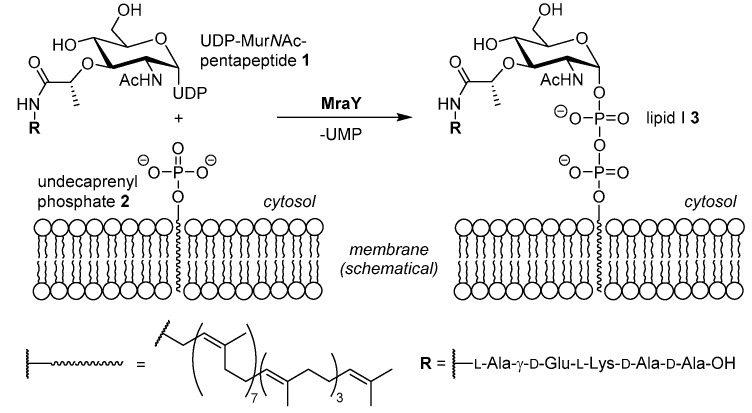
The reaction mediated by the bacterial membrane enzyme MraY (translocase I). UDP = uridine diphosphate, UMP = uridine monophosphate. The exact composition of the pentapeptide unit (residue **R**) can vary among different bacteria [12].

Bacterial infections with pathogens resistant against established antimicrobial drugs are an emerging concern in current and future healthcare [1,2]. In order to address this issue, new antibacterial drug candidates are urgently needed. These should ideally display new or previously unexploited modes of action to avoid cross resistance with existing drugs. One way to reach this goal might be the identification of new targets within bacterial pathways which are blocked by existing antibiotics [3]. Inhibition of the formation of the bacterial cell wall, i.e., of peptidoglycan biosynthesis, is a highly attractive mode of action for antimicrobial drugs as there is no human counterpart to this bacterial process [3,4]. Therefore, inhibitors of peptidoglycan biosynthesis can be anticipated to be highly selective with very limited toxicity to human host cells. Within peptidoglycan biosynthesis, the bacterial membrane enzyme MraY (translocase I) represents a hitherto unexploited target [5,6]. MraY mediates the first membrane-associated step of the intracellular section of peptidoglycan formation. Thus, it catalyses the reaction of uridine diphosphate *N*-acetylmuramic acid (UDP-Mur*N*Ac) pentapeptide (‘Park’s nucleotide’) **1** with the isoprenoid membrane anchor undecaprenyl phosphate **2** to give membrane-bound lipid I **3** (Scheme 1) [7,8,9,10,11,12].

**Figure 1:**
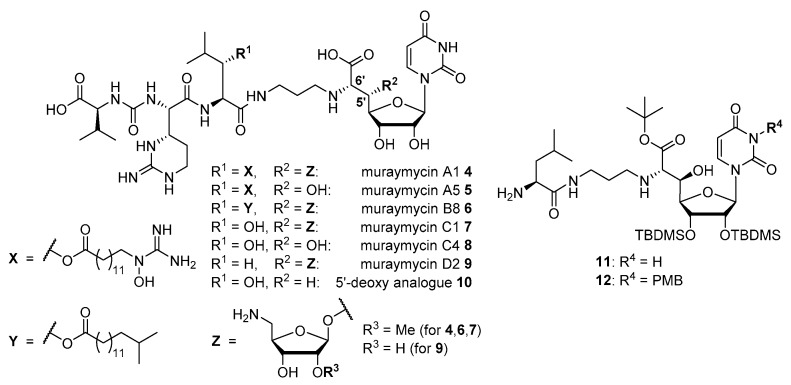
Selected naturally occurring muraymycins **4**–**9** [17,18], the previously reported synthetic 5′-deoxy analogue **10** [19] and other previously reported muraymycin analogues **11** and **12** [20].

A large family of antibacterially active natural products is known to inhibit MraY. These microbial secondary metabolites have in common that they are uridine-derived nucleoside analogues, and hence, they are often referred to as ‘nucleoside antibiotics’. These nucleoside antibiotics are divided into a range of structurally different subclasses, e.g., muraymycins, caprazamycins, liposidomycins, mureidomycins, pacidamycins, capuramycins and tunicamycins [13,14,15,16]. We have set up a long-term research program on muraymycins and their synthetic analogues in order to develop them into potential antibacterial drug candidates. Muraymycins were originally reported as a collection of 19 structurally related secondary metabolites from *Streptomyces* [17]. Just recently, some previously unknown members of the muraymycin subclass were isolated from the producing strains [18]. The muraymycin scaffold consists of a (5′*S*,6′*S*)-glycyluridine (GlyU) unit (i.e., a so-called ‘high-carbon’ nucleoside) and a urea peptide moiety, both connected by a short alkyl linker. The urea peptide contains the non-proteinogenic amino acid epicapreomycidine as a cyclic arginine derivative (Figure 1). Most of the naturally occurring muraymycins are 5′-*O*-aminoribosylated in the GlyU core unit (structure **Z** in Figure 1).

Muraymycins have been formally divided into four subclasses based on the exact structure of the central l-leucine unit: subclasses A-C contain a (3*S*)-3-hydroxyl-l-leucine that is derivatised with fatty acyl moieties in the A- and B-series. In the A-series, one can find ω-substituted fatty acyl structures with a terminal (*N*-hydroxyl-)guanidine group (e.g., muraymycins A1 **4** and A5 **5**, Figure 1). In contrast, the B-series contains branched alkyl chains in this position (e.g., muraymycin B8 **6**). The C-series (e.g., muraymycin C1 **7** and C4 **8**) is not *O*-acylated at the 3-hydroxyl-l-leucine, and the D-series (e.g., muraymycin D2 **9**) contains proteinogenic non-hydroxylated l-leucine at this position.

Methodology for the total synthesis of muraymycins has been developed [15], even though synthetic access to the 5′-*O*-aminoribosylated ‘high-carbon’ GlyU core structure as well as the epicapreomycidine and the (3*S*)-3-hydroxyl-l-leucine units is challenging. Relevant contributions to this field have been reported by Ichikawa and Matsuda [21,22], Kurosu [23,24], and our group [25,26,27,28]. Structure-activity relationship (SAR) studies on muraymycin analogues have already been performed and also included structurally simplified derivatives [15,19,20,29,30,31,32,33,34]. Some of these findings are subsequently highlighted. Our group has described 5′-defunctionalised (‘5′-deoxy’) analogues of the uridine-derived GlyU core unit, i.e., derivatives not only lacking the aminoribosyl moiety (**Z** in Figure 1), but any substituent in the 5′-position [19,34,35,36]. For instance, in case of the 5′-deoxy analogue **10** of muraymycin C4 **8** (Figure 1), it was shown that the structurally simplified muraymycin congener **10** still was a rather potent inhibitor of MraY in vitro (IC_50_ = 95 ± 19 nM for **10**) [19,33]. Shortly after the isolation of the naturally occurring muraymycins, Yamashita et al. reported the synthesis and biological activity of truncated 5′-*epi*-muraymycin analogues **11** and **12** (Figure 1) [20]. These compounds were found to inhibit the growth of some Gram-positive pathogens in cellulo and MraY in vitro (using a coupled MraY-MurG assay). This was remarkable as **11** and **12** still contained synthetic protecting groups, i.e., *para*-methoxybenzyl (PMB) at the uracil*-N-*3 (only for **12**), *tert*-butyldimethylsilyl (TBDMS) at 2′-O and 3′-O, and a *tert*-butyl ester moiety. Deprotection of the compounds led to a loss of bioactivity. Recently, we have re-investigated some naturally occurring muraymycins for their activities as MraY inhibitors [33]. We demostrated that the absence of the fatty acid moiety (such as in **7**–**9**) leads to a loss of antimicrobial activity in cellulo (most likely due to impaired cellular uptake of the non-lipophilised muraymycins), but retention of MraY inhibition in vitro. The 5′-*O*-aminoribosylated muraymycin natural products were found to be extremely potent MraY inhibitors, with IC_50_ values in the pM range [33].

MraY is an integral membrane protein, which makes its overexpression as well as structural biology studies challenging. Nevertheless, methodology for the overexpression and isolation of MraY has been described [37,38,39]. For in vitro assays of MraY activity and inhibition, an efficient fluorescence-based method using a dansylated derivative of Park’s nucleotide **1** is established [33,34,40,41,42,43]. Based on an in silico topology model, it had been predicted that MraY would be composed of ten transmembrane helices and five cytosolic loops [44]. This proposed architecture was experimentally confirmed when Lee reported the first X-ray crystal structure of MraY from the extremophile *Aquifex aeolicus* [45]. Following this structure of ligand-free MraY, the same group accomplished the first X-ray crystal structure of MraY (again from *Aquifex aeolicus*) in complex with an inhibitor, i.e., with muraymycin D2 **9** (PDB 5CKR, 2.95 Å resolution) [46,47]. A comparison of this co-crystal structure with the structure of the aforementioned ligand-free *apo* enzyme [45] revealed a major conformational change upon inhibitor binding, i.e., a significant conformational plasticity of the enzyme. Another co-crystal structure of MraY from *C. bolteae* in complex with the nucleoside antibiotic tunicamycin has also been reported [48].

The conformational plasticity of MraY (vide supra) and the complex structures of nucleoside antibiotics make computer-aided design for the development of new nucleosidic MraY inhibitors very challenging. We have managed to obtain proposed binding modes for several naturally occurring muraymycins by in silico modelling, based on the co-crystal structure of MraY with muraymycin D2 **9** [33]. However, it is precluded to apply such a procedure for synthetic muraymycin analogues with more pronounced structural differences to natural product **9**.

With respect to the complex and synthetically challenging structures of naturally occurring muraymycins, one general long-term goal of our research is to identify bioactive, structurally simplified muraymycin analogues with improved chemical tractability. In this context, we would like to utilise the insights provided by the co-crystal structure of MraY with muraymycin D2 **9**. As in silico modelling on this system is associated with major hurdles (vide supra), we aim to derive information from the co-crystal structure that inspires the design of novel muraymycin analogues. Subsequent synthesis and biological testing can then probe the initial proposal. In this work, we report on one example of such an approach, i.e., the design, synthesis and biological evaluation of aminoribosylated, but structurally simplified muraymycin analogues.

## 2. Results

### 2.1. Design of Target Structures

**Figure 2:**
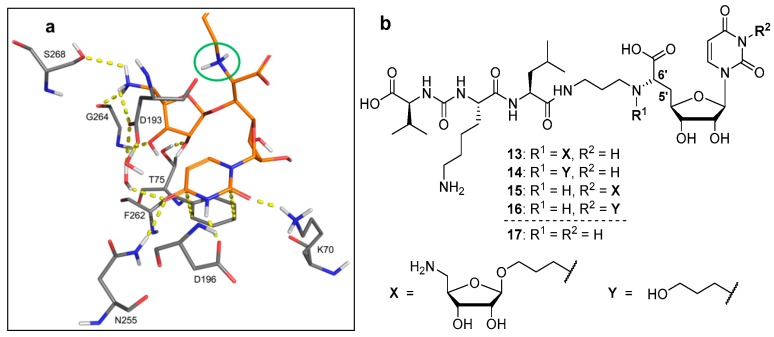
(**a**) Insights into the X-ray co-crystal structure of MraY from *Aquifex aeolicus* in complex with muraymycin D2 **9** (PDB 5CKR) [46,47]: protein-inhibitor interactions at the nucleoside binding site and its close proximity (hydrocarbon scaffold of inhibitor **9** in orange, green circle highlights 6′-N). (**b**) Target structures **13–16** of this study with previously reported muraymycin analogue **17** [34].

For the design of the target structures of this study, we have inspected the co-crystal structure of MraY from *Aquifex aeolicus* in complex with the inhibitor muraymycin D2 **9** [46,47] more closely. This particularly concerned the interaction of the 5′-*O*-aminoribosylated GlyU core unit of **9** with MraY. The complex reveals a fairly specific set of interactions for the aminoribose moiety, including a network of hydrogen bonds and an electrostatic interaction of the ribose-5″-amino group with Asp-193 (Figure 2a). This finding is in line with the significant contribution of the aminoribose unit to MraY inhibition: while 5′-*O*-aminoribosylated naturally occurring muraymycins were pM inhibitors of MraY in vitro, inhibitory potency dropped to the nM range for 5′-defunctionalised synthetic analogue **10** (vide supra) [33]. Another very specific set of interactions can be found for the uracil nucleobase, which is accommodated in a pocket with hydrogen bonding (with Asn-255, Asp-196, Lys-70) and π-stacking (with Phe-262).

In this work, our principle approach was to attempt a different linkage of the aminoribosyl unit to the muraymycin scaffold, i.e., a connection not via the 5′-hydroxyl group. Synthetic access to the 5′-aminoribosylated GlyU core unit is not trivial and involves multi-step routes [22,24,25,49,50,51,52]. Ideally, the novel aminoribosylated analogues would combine an improved chemical tractability with strong MraY inhibitory potencies due to interactions of the aminoribose motif with the protein. These considerations have led to the design of target structures **13**–**16** (Figure 2b), which were derived from the previously reported simplified muraymycin analogue **17** [34]. In **17**, the epicapreomycidine unit is replaced with proteinogenic l-lysine, the central l-leucine is not β-hydroxylated (as in muraymycin D2 **9**), and the 5′-defunctionalised version of the GlyU core is employed (as in analogue **10**, vide supra). This design had given a moderate, but notable inhibitory activity of **17** towards MraY from *S. aureus* (IC_50_ = 2.5 ± 0.6 μM) [34]. In this proof-of-principle study, we aimed to investigate if chemically tractable muraymycin analogue **17** can be decorated with the aminoribose unit, connected to the scaffold via a linker.

A connection site at the muraymycin scaffold in fair proximity to the native 5′-position was preferable. Therefore, we decided to link the aminoribose moiety to the 6′-amino group of the 5′-deoxy-GlyU core. The X-ray co-crystal structure revealed the protonated 6′-amino group of the inhibitor to be oriented in a way that the two hydrogen atoms point towards the α-face of the β-configured aminoribose unit (Figure 2a). It was estimated that a short alkyl linker might be sufficient for bridging the distance to the β-face of the aminoribose moiety. Hence, the connection of the aminoribose to the 6′-amino group was attempted with a three-carbon linker, thus leading to the first 6′-substituted target structure **13** (Figure 2b). As a reference compound, we planned to also synthesise **14**, which contains the 6′-linker unit (terminated with a polar hydroxyl group), but not the aminoribose motif. In search for an alternative attachment site at the muraymycin scaffold, we then considered the uracil nucleobase. The highly specific uracil binding pocket of MraY (vide supra) seems to preclude functionalisation of the uracil base. On the other hand, uracil*-N-*3-*p*-methoxybenzylated analogue **12** (see Figure 1) had been reported to be an MraY-inhibiting antibiotic (vide supra) [20]. We speculated that this might be a consequence of a slightly different binding mode of *N*-3-substituted muraymycin derivatives to MraY, which would explain the apparent contradiction to the insights from the X-ray co-crystal structure. In order to probe this hypothesis, we have therefore designed uracil*-N-*3-substituted target structure **15** (Figure 2b). Again, the three-carbon linker was envisioned to potentially bridge the distance to the aminoribose binding pocket. As for the first target structure, a non-aminoribosylated reference compound **16** with the attached linker motif was also planned to be synthesised and evaluated.

### 2.2. Synthesis of Aminoribosylated Muraymycin Analogues and Reference Compounds

**Scheme 2:**
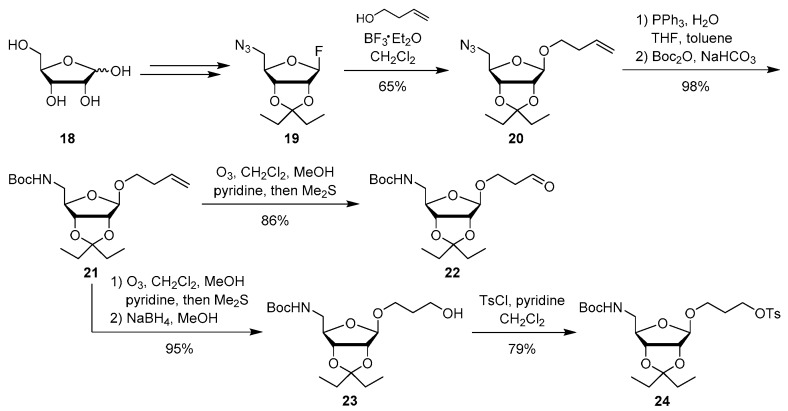
Synthesis of aminoribosyl-linker building blocks **22** and **24**.

For the synthesis of aminoribosylated target compounds **13** and **15**, suitably protected aminoribose-containing building blocks were required. Starting from d-ribose **18**, we therefore prepared anomerically pure 2,3-pentylidene-protected 5-azido-5-deoxy-β-ribosyl fluoride **19** according to established procedures (Scheme 2) [49,50,51,52]. With **19** as a glycosyl donor and homoallylic alcohol as acceptor, riboside **20** was obtained in 65% yield. The pronounced β-selectivity resulted from steric shielding of the α-face [49,50]. A sequence of Staudinger reduction of the azide and Boc protection then afforded butenyl riboside **21** in 98% yield.

Ozonolysis of the double bond in **21** furnished aldehyde **22** (86% yield, Scheme 2) as a reagent for the introduction of the aminoribosyl-linker unit by reductive amination. For an alternative attachment of the aminoribosyl-linker moiety by nucleophilic substitution, butenyl riboside **21** was converted in a different manner. It also underwent ozonolysis, but the resultant aldehyde was not purified and directly reduced to alcohol **23** in 95% yield. Tosylation of the hydroxyl group then gave **24** (79% yield) as a reactive derivative for nucleophilic substitions.

**Scheme 3:**
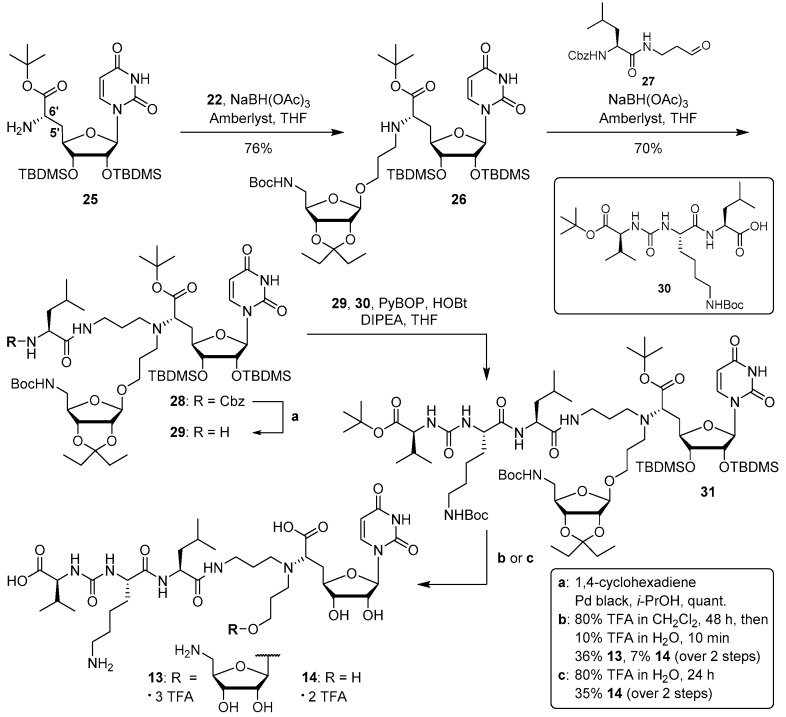
Synthesis of target structures **13** and **14**.

For the synthesis of target compound **13**, we started the construction of the muraymycin scaffold from the diastereomerically pure, protected (6′*S*)-5′-deoxy nucleosyl amino acid **25**, which had been prepared from uridine according to previously reported protocols [35,36]. A reductive amination reaction of aldehyde **22** with uridine derivative **25** gave the *N*-alkylated product **26** in 76% yield (Scheme 3). Another reductive amination of **26** with l-leucine-derived aldehyde **27** (which had been prepared as described before [20]) then furnished tertiary amine **28** in 70% yield. Remarkably, the order of the two reductive aminations (first with **22**, then with **27**) was crucial. Attempts to reverse the order, i.e., to perform the reductive amination with aldehyde **27** first, resulted in a failure of the second reductive amination reaction. We speculate that this might be owed to increased steric hindrance of the 6′-amino group in the latter case.

The synthesis of target structure **13** was completed in the following manner. Hydrogenolysis of the Cbz protecting group in **28** gave primary amine **29** in quantitative yield (Scheme 3). Coupling of **29** with the urea dipeptide building block **30** (which had been synthesised as reported before [34]) was accomplished with (benzotriazol-1-yloxy)tripyrrolidinophosphonium hexafluorophosphate (PyBOP) and 1-hydroxybenzotriazole (HOBt) to afford the protected full-length muraymycin analogue **31**. This compound was directly subjected to global acidic deprotection. Surprisingly, usual standard conditions for this transformation (80% trifluoroacetic acid (TFA) in water [19,22,31,34,36]) did not only lead to the desired removal of all protecting groups, but also to the hydrolysis of the glycosidic bond at the aminoribose moiety. We assume that the reduced steric hindrance at the anomeric center of the aminoribosyl unit, relative to 5′-*O*-aminoribosylated muraymycins, might account for this unexpected hurdle. In order to synthesise **13**, we carefully optimised this acidic deprotection step, including various concentrations of TFA and different solvents. Best results were achieved using 80% TFA in dichloromethane for 48 h, followed by 10% TFA in water for just 10 min. This protocol gave, after purification by semipreparative HPLC, **13** (as tris-TFA salt) in 36% yield and reference compound **14** (as bis-TFA salt) in 7% yield, respectively, each over two steps from **29**. A more efficient way to obtain non-aminoribosylated reference **14** was the treatment of **31** with 80% aqueous TFA, which afforded **14** in 35% yield over two steps from **29** (Scheme 3).

**Scheme 4:**
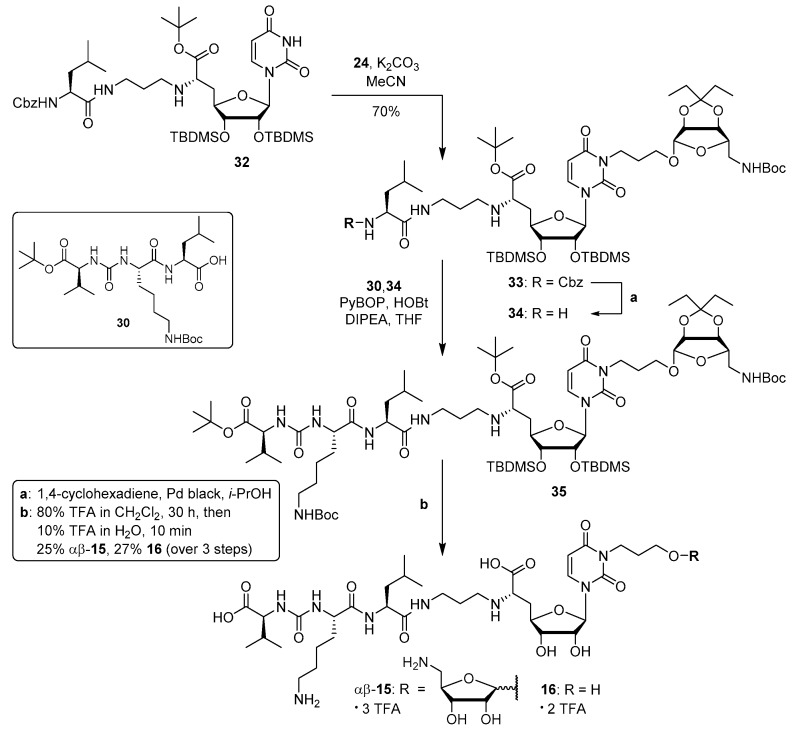
Synthesis of target structures **15** and **16**.

For the synthesis of target compound **15**, we employed the previously reported protected 5′-deoxy muraymycin building block **32** [36] as starting material. Selective alkylation of **32** with tosylate **24** at the uracil-*N*-3-position was achieved in 70% yield (product **33**, Scheme 4). The position of the newly introduced aminoribosyl-linker unit was unambigously proven by 2D NMR spectroscopy. The pronounced regioselectivity of this reaction (uracil*-N-*3 vs. 6′-amino group) was probably owed to the significant steric shielding of the electronically more nucleophilic 6′-amino functionality.

Hydrogenolysis of the Cbz protecting group in **33** then gave primary amine **34**, which was used in the next step in crude form. Coupling of **34** with the urea dipeptide building block **30** [34] was achieved with PyBOP and HOBt to afford the protected full-length muraymycin analogue **35**. This compound was directly subjected to global acidic deprotection. Again, a range of conditions was studied in order to control the potential hydrolytic cleavage of the aminoribosyl unit. Best results were obtained using 80% TFA in dichloromethane for 30 h, followed by 10% TFA in water for just 10 min. This procedure gave, after purification by semipreparative HPLC, **15** (as tris-TFA salt) in 25% yield and reference compound **16** (as bis-TFA salt) in 27% yield, respectively, each over three steps from **33**. Remarkably, the acidic deprotection also led to epimerisation at the anomeric center of the aminoribosyl moiety, thus furnishing an inseparable 1:1 mixture of the α- and the β-anomer. Hence, this compound is from now on referred to as αβ-**15** (Scheme 4). At this stage, it was decided not to attempt a stereocontrolled synthesis of **15**, but to evaluate the properties of αβ-**15** as an MraY inhibitor first.

### 2.3. Biological Evaluation

All target structures **13**–**16** were tested in vitro as potential inhibitors of the bacterial target enzyme MraY. For this, we employed the fluorescence-based assay for MraY activity (vide supra), which utilises a dansylated derivative of Park’s nucleotide **1** and MraY from *S. aureus* (recombinantly overexpressed in *E. coli*) [33,34,40,41,42,43]. None of the tested muraymycin analogues showed interfering autofluorescence at the used excitation wavelength (λ_ex_ = 355 nm). The obtained inhibitory activities (IC_50_ values) are listed in Table 1. Muraymycin analogues **13**–**16** were also investigated for their antibacterial activities in cellulo against *E. coli*, but turned out not to be active (MIC > 50 μg/mL).

## 3. Discussion

We have accomplished the synthesis of the four envisioned target muraymycin analogues **13**–**16** in an efficient manner, even though **15** was obtained as an inseparable 1:1 mixture of anomers (αβ-**15**). Strategies for the regioselective introduction of an aminoribosyl-linker substituent at either the 6′-amino group or the uracil*-N-*3 were developed. One unexpected finding was the hydrolytic lability of the aminoribose glycosidic bond under acidic deprotection conditions which had been successful for 5′-*O*-aminoribosylated muraymycins. This probably correlated with a reduced steric hindrance at the aminoribosyl-linker motif. However, this hurdle could be utilised for an efficient synthesis of the two non-aminoribosylated analogues **14** and **16**. Both en route to **13** and **15**, acidic deprotection conditions could be adjusted in a way that enabled the isolation of the aminoribosylated target structures as well as **14** and **16**, thus avoiding separate synthetic routes towards these two reference compounds. In the synthesis of αβ-**15**, the higher amount of deribosylated product **16** formed in the acidic deprotection step and the epimerisation at the anomeric center indicated that the aminoribosyl unit in **15** was probably even less sterically shielded than the corresponding structural motif in 6′-substituted congener **13**.

The in vitro evaluation of **13–16** as MraY inhibitors revealed that only reference compound **14** showed notable inhibitory potency in the relevant concentration range (<100 μM). With an IC_50_ value of 17 ± 9 μM, it was just slightly less active than the previously reported muraymycin analogue **17** (IC_50_ = 2.5 ± 0.6 μM) [34]. The non-activity of 6′-substituted aminoribosylated analogue **13** indicated that the design concept inspired by the X-ray co-crystal structure, i.e., a bridging of the aminoribose unit to the 6′-position, did not work out. However, the activity of **14** demonstrated that a substitution of the 6′-position towards a tertiary amine does not necessarily lead to a major loss of activity. It is possible that the selected three-carbon linker is not ideal to place the 6′-connected aminoribose unit in the corresponding binding pocket of MraY. It has to be emphasised that a more exact in silico modelling of an approriate linker architecture is hampered by the pronounced conformational plasticity of MraY and the size and conformational flexibility of muraymycin-derived inhibitors. This leaves the identification of suitable linkers to experimental approaches such as the one reported in this work.

The inactivity of both **15** and **16** confirmed what had been derived from the X-ray co-crystal structure, i.e., that a substitution at the uracil*-N-*3 is precluded by the highly specific interaction of this motif with MraY. While this effect might be assigned to steric bulk of the substituent in the case of **15**, the non-activity of **16** (bearing a much smaller N-3 substituent) suggests that any derivatisation of the uracil*-N-*3 might lead to a loss of inhibitory activity. This is in contrast to the reported bioactivity of muraymycin analogue **12** (see Figure 1) [20] though. It cannot be ruled out that **12** might bind to MraY in a different binding mode, but this remains to be studied in future work. However, it was our goal to probe the concept of uracil*-N-*3 derivatisation, and this aim was successfully accomplished as the reported results generally discourage uracil*-N-*3 substitution.

As target structures **13**, **15** and **16** did not show notable potency as MraY inhibitors, it was not surprising that they were also antibacterially inactive in cellulo against *E. coli*. In the case of MraY-inhibiting analogue **14**, the missing antibacterial activity requires further discussion. We had found out in our previous work that there might be a ‘threshold’ of inhibitory potency towards MraY for observing antibacterial activity in cellulo [34]. For instance, analogue **10** (as a nM MraY inhibitor) shows some rather moderate activity against the growth of *E. coli* [19], while **17** (as a low-μM MraY inhibitor) was inactive against the same strains [34]. From these observations, we had concluded that a low-nM (or stronger) inhibitory potency against MraY seems to be a prerequisite for reasonable antibacterial activity [34]. As target compound **14** was a slightly weaker MraY inhibitor than **17**, it is not surprising that it was devoid of antibacterial activity. It needs to be taken into account though that the polar muraymycin scaffold impairs cellular uptake, thus making the penetration of the bacterial membrane a bottleneck for antibacterial activity. This hurdle might be overcome, for instance, by lipophilisation of the polar muraymycin structure. However, it should be pointed out that the primary goal of this study was to further elucidate MraY inhibition and not to obtain novel analogues with strong antibacterial potencies.

## 4. Materials and Methods

### 4.1. Synthesis of Aminoribosylated Muraymycin Analogues and Reference Compounds

General Methods: All chemicals were purchased from standard suppliers. Reactions involving oxygen and/or moisture sensitive reagents were carried out under an atmosphere of argon using anhydrous solvents. Anhydrous solvents were obtained in the following manner. CH_2_Cl_2_, MeCN and THF were purchased in HPLC quality, dried with a solvent purification system (MBRAUN MB SPS 800) and stored over activated molecular sieves (3 Å (CH_2_Cl_2_, MeCN) or 4 Å (THF)). Pyridine was dried over CaH_2_, distilled and stored over activated molecular sieves (4 Å). MeOH was degassed, dried and stored over activated molecular sieves (3 Å). *i*-PrOH was dried over calcium sulfate hemihydrate and stored over activated molecular sieves (3 Å). All other solvents were of technical quality and distilled prior to use, and deionised water was used throughout. Column chromatography was carried out on silica gel 60 (0.040–0.063 mm, 230–400 mesh ASTM, VWR, Darmstadt, Germany) under flash conditions except where indicated. TLC was performed on aluminium plates precoated with silica gel 60 F_254_ (VWR, Darmstadt, Germany). Visualisation of the spots was carried out using UV light (254 nm) and/or staining under heating (H_2_SO_4_ staining solution: 4 g vanillin, 25 mL conc. H_2_SO_4_, 80 mL AcOH, and 680 mL MeOH; KMnO_4_ staining solution: 1 g KMnO_4_, 6 g K_2_CO_3_, and 1.5 mL 1.25 M NaOH solution, all dissolved in 100 mL H_2_O; ninhydrin staining solution: 0.3 g ninhydrin, 3 mL AcOH, and 100 mL 1-butanol). Analytical HPLC was performed on a Thermo Scientific system (ThermoFisher Scientific, Dreieich, Germany) equipped with an MWD detector (250.0), an ESI mass spectrometer and an EC 125/3 column (5 × 100 mm) containing reversed phase silica gel NUCLEODUR^TM^ 100–5 C18ec (5 μm, Macherey-Nagel, Düren, Germany). Method 1: eluent A water (+0.1% TFA), eluent B MeCN (+0.1% TFA); 0–19 min gradient of B (5–100%), 19–22 min 100% B, 22–23 min gradient of B (100–5%), 23–25 min 5% B; flow 0.8 mL/min. Semipreparative HPLC was performed on an Agilent Technologies 1200 Series system (Agilent Technologies, Waldbronn, Germany) equipped with an MWD detector (254.16/280.16) and a LiChroCart^TM^ column (10 × 250 mm) containing reversed phase silica gel Purospher^TM^ RP18e (5 μm, VWR). Method 1: eluent A water (+0.1% TFA), eluent B MeCN (+0.1% TFA); 0–36 min gradient of B (5–100%), 36–44 min 100% B, 44–44.1 min gradient of B (100–1%); flow 3 mL/min. Method 2: eluent A water (+0.1% TFA), eluent B MeCN (+0.1% TFA); 0–36 min gradient of B (5–60%), 36–40 min gradient of B (60–100%), 40–44 min 100% B, 44–44.1 min gradient of B (100–1%); flow 3 mL/min. Method 3: eluent A water (+0.1% TFA), eluent B MeCN (+0.1% TFA); 0–36 min gradient of B (5–70%), 36–40 min gradient of B (70–100%), 40–44 min 100% B, 44–44.1 min gradient of B (100–1%); flow 3 mL/min. 500 MHz-^1^H, 126 MHz-^13^C, as well as 282 MHz-^19^F NMR spectra were recorded on Bruker AVANCE-500 and AVANCE-300 spectrometers (Bruker, Bremen, Germany). All ^13^C and ^19^F NMR spectra are ^1^H-decoupled. All spectra were recorded at room temperature except where indicated otherwise and were referenced internally to solvent reference frequencies. Chemical shifts (δ) are quoted in ppm and coupling constants (*J*) are reported in Hz. Assignment of signals was carried out using H,H-COSY, HSQC, and HMBC spectra obtained on the spectrometers mentioned above. The numbering of atoms of muraymycin target structures is depicted in the Supplementary Materials (Figure S1). Mass spectra of small molecules were measured on a Finnigan LCQ ion-trap mass spectrometer or on a Bruker microTOF spectrometer (Bruker, Bremen, Germany). High resolution spectra were measured on a Bruker 7 Tesla Fourier transform ion cyclotron resonance (FTICR) mass spectrometer (Bruker, Bremen, Germany). Infrared spectroscopy (IR) was performed on a Bruker Vertex 70 spectrometer equipped with an integrated ATR unit (PlatinumATR^TM^, Bruker). Wavenumbers (ν) are quoted in cm^−1^. UV spectroscopy was carried out on an Cary 100 spectrophotometer (Agilent Technologies, Waldbronn, Germany). Wavelengths of maximum absorption (λ_max_) are reported in nm. Optical rotations were recorded on a Krüss Optronic polarimeter with a Na source using a 5 cm cell (Krüss Optronic, Hamburg, Germany). Melting points (m.p.) were measured on a Büchi instrument (Büchi, Essen, Germany) and are not corrected.

6′*-N-*Substituted aminoribosylated muraymycin analogue (**13**): To a solution of protected urea dipeptide **30** (6.1 mg, 14 μmol) in THF (2.5 mL), HOBt (1.9 mg, 14 μmol), PyBOP (7.1 mg, 14 μmol) and DIPEA (4.7 μL, 27 μmol) were added and the mixture was stirred at rt for 30 min. At 0 °C, a solution of protected 6′*-N-*substituted aminoribosylated truncated muraymycin analogue **29** (15.2 mg, 13.7 μmol) in THF (2 mL) was added dropwise, and the reaction mixture was stirred at 0 °C for 1 h and at rt for 3 h. The solvent was evaporated under reduced pressure, and the resultant crude product was purified by column chromatography (97:3, CH_2_Cl_2_-MeOH) to give **31** as a colourless solid (20.4 mg) that was directly used in the subsequent deprotection step. A solution of the protected 6′*-N-*substituted aminoribosylated muraymycin analogue **31** (16.0 mg) in TFA (80% in CH_2_Cl_2_, 5.0 mL) was stirred at rt for 48 h. The mixture was then diluted with CH_2_Cl_2_ (20 mL) and the solvent was evaporated under reduced pressure. The resultant residue was dissolved in TFA (10% in water, 5.0 mL), the mixture was stirred at rt for 10 min and then diluted with water (20 mL). The solvent was evaporated under reduced pressure, and the resultant crude product was purified by semipreparative HPLC (method 1) to give **13** (tris-TFA salt) as a colourless solid (5.0 mg, 36% over 2 steps from **29**) and **14** (bis-TFA salt) as a colourless solid (0.75 mg, 7% over 2 steps from **29**). **13**: ^1^H NMR (500 MHz, D_2_O): δ [ppm] = 0.90 (d, *J* = 6.1 Hz, 3 H, 5‴-H_a_), 0.95 (d, *J* = 5.2 Hz, 3H, 5‴-H_b_), 0.96 (d, *J* = 6.2 Hz, 3H, 4^v^-H_a_), 1.00 (d, *J* = 6.8 Hz, 3H, 4^v^-H_b_), 1.41–1.54 (m, 2H, 4^iv^-H), 1.56–1.77 (m, 6H, 3‴-H, 4‴-H, 3^iv^-H_a_, 5^iv^-H), 1.70–1.86 (m, 1H, 3^iv^-H_b_), 1.95–2.06 (m, 3H, 2″-H, 2^vi^-H_a_), 2.06–2.15 (m, 1H, 2^vi^-H_b_), 2.16–2.25 (m, 2H, 5′-H_a_, 3^v^-H), 2.43–2.49 (m, 1H, 5′-H_b_), 3.03 (dd, *J* = 7.6, 7.6 Hz, 2H, 6^iv^-H), 3.09 (dd, *J* = 11.5, 11.1 Hz, 1H, 5^vii^-H_a_), 3.16–3.27 (m, 2H, 1″-H_a_, 3″-H_a_), 3.27–3.34 (m, 2H, 1″-H_b_, 1^vi^-H_a_), 3.34–3.40 (m, 2H, 3″-H_b_, 1^vi^-H_b_), 3.42 (dd, *J* = 13.3, 2.4 Hz, 1H, 5^vii^-H_b_), 3.61–3.67 (m, 1H, 3^vi^-H_a_), 3.88 (ddd, *J* = 6.9, 4.3, 3.9 Hz, 1H, 3^vi^-H_b_), 4.07 (dd, *J* = 9.6, 3.1 Hz, 1H, 6′-H), 4.10–4.19 (m, 5H, 3′-H, 2^iv^-H, 2^v^-H, 2^vii^-H, 4^vii^-H), 4.22 (dd, *J* = 6.3, 4.8 Hz, 1H, 3^vii^-H), 4.26 (dd, *J* = 7.7, 6.6 Hz, 1H, 4′-H), 4.30 (dd, *J* = 10.0, 4.7 Hz, 1H, 2‴-H), 4.55 (dd, *J* = 4.9, 4.5 Hz, 1H, 2′-H), 5.03 (s, 1H, 1^vii^-H), 5.76 (d, *J* = 4.5 Hz, 1H, 1′-H), 5.92 (d, *J* = 8.1 Hz, 1H, 5-H), 7.69 (d, *J* = 8.1 Hz, 1H, 6-H). ^13^C NMR (126 MHz, D_2_O): δ [ppm] = 16.96 (C_a_-4^v^), 18.49 (C_b_-4^v^), 20.58 (C_a_-5‴), 21.95 (C-4^iv^), 22.12 (C_b_-5‴), 24.13 (C-2″), 24.19 (C-2^vi^), 24.40 (C-4‴), 26.25 (C-5^iv^), 29.46 (C-5′), 29.92 (C-3^v^), 30.68 (C-3^iv^), 36.14 (C-3″), 39.21 (C-6^iv^), 39.52 (C-3‴), 43.11 (C-5^vii^), 49.65 (C-1″), 50.64 (C-1^vi^), 52.47 (C-2‴), 54.24 (C-2^iv^), 58.89 (C-2^v^), 63.30 (C-6′), 65.62 (C-3^vi^), 72.36 (C-3^vii^), 72.42 (C-2′), 73.08 (C-3′), 74.05 (C-2^vii^), 78.26 (C-4^vii^), 80.52 (C-4′), 92.19 (C-1′), 102.29 (C-5), 107.52 (C-1^vii^), 116.33 (q, ^1^*J*_CF_ = 291.5 Hz, F_3_CCOO), 143.23 (C-6), 151.28 (C-2), 159.51 (NC(=O)N), 162.97 (q, ^2^*J*_CF_ = 35.5 Hz, F_3_CCOO), 166.09 (C-4), 171.57 (C-7′), 174.76 (C-1‴), 175.45 (C-1^iv^), 176.71 (C-1^v^). ^19^F NMR (282 MHz, D_2_O): δ [ppm] = −75.57 (s, CF_3_). HRMS (ESI^+^): calcd.: 466.7504 [M+2H]^2+^, found: 466.7504. IR (ATR): *ν* = 3066, 2962, 1668, 1556, 1464, 1199, 1131, 1030, 835, 799, 721, 551. UV (MeCN): λ_max_ = 260. optical rotation: [α]_D_^20^ = −11.3 (c = 1.3, H_2_O). m.p. = 145 °C. Analytical HPLC: *t*_R_ = 6.7 min (method 1). Semipreparative HPLC: *t*_R_ = 12.3 min (method 1). **14**: see below for analytical data.

6′*-N-*Substituted non–aminoribosylated muraymycin analogue (**14**): Protected 6′*-N-*substituted aminoribosylated muraymycin analogue **31** was prepared as described above. A solution of **31** (21.2 mg) in TFA (80% in water, 6.0 mL) was stirred at rt for 24 h. The mixture was then diluted with water (20 mL) and the solvent was evaporated under reduced pressure. The resultant crude product was purified by semipreparative HPLC (method 2) to give **14** (tris-TFA salt) as a colourless solid (5.1 mg, 35% over 2 steps from **29**). ^1^H NMR (500 MHz, D_2_O): δ [ppm] = 0.80 (d, *J* = 6.1 Hz, 3H, 5‴-H_a_), 0.85 (d, *J* = 4.9 Hz, 3H, 5‴-H_b_), 0.86 (d, *J* = 6.1 Hz, 3H, 4^v^-H_a_), 0.90 (d, *J* = 6.7 Hz, 3H, 4^v^-H_b_), 1.31–1.43 (m, 2H, 4^iv^-H), 1.46–1.67 (m, 6H, 3‴-H, 4‴-H, 3^iv^-H_a_, 5^iv^-H), 1.69–1.76 (m, 1H, 3^iv^-H_b)_, 1.84–1.95 (m, 4H, 2″-H, 2^vi^-H), 2.10 (dqq, *J* = 6.7, 6.6, 6.1 Hz, 1H, 3^v^-H), 2.14 (ddd, *J* = 14.0, 10.7, 3.0 Hz, 1H, 5′-H_a_), 2.37–2.42 (m, 1H, 5′-H_b_), 3.03 (dd, *J* = 7.5, 7.5 Hz, 2H, 6^iv^-H), 3.07–3.19 (m, 2H, 1″-H_a_, 3″-H_a_), 3.19–3.27 (m, 3H, 1″-H_b_, 3″-H_b_, 1^vi^-H_a_), 3.29–3.35 (m, 1H, 1^vi^-H_b_), 3.60–3.68 (m, 2H, 3^vi^-H), 4.00–4.10 (m, 4H, 3′-H, 6′-H, 2^iv^-H, 2^v^-H), 4.15–4.22 (m, 1H, 4′-H, 2‴-H), 4.44 (dd, *J* = 5.1, 4.4 Hz, 1H, 2′-H), 5.67 (d, *J* = 4.4 Hz, 1H, 1′-H), 5.82 (d, *J* = 8.1 Hz, 1H, 5-H), 7.59 (d, *J* = 8.1 Hz, 1H, 6-H). ^13^C NMR (126 MHz, D_2_O): δ [ppm] = 16.94 (C_a_-4^v^), 18.47 (C_b_-4^v^), 20.61 (C_a_-5‴), 22.01 (C-4^iv^), 22.09 (C_b_-5‴), 24.22 (C-2″), 24.38 (C-4‴), 26.21 (C-2^vi^), 26.24 (C-5^iv^), 29.34 (C-5′), 29.92 (C-3^v^), 30.74 (C-3^iv^), 36.11 (C-3″), 39.21 (C-6^iv^), 39.54 (C-3‴), 49.87 (C-1″), 50.97 (C-1^vi^), 52.50 (C-2‴), 54.08 (C-2^iv^), 58.84 (C-2^v^), 59.13 (C-3^vi^), 62.70 (C-6′), 72.47 (C-2′), 73.02 (C-3′), 80.50 (C-4′), 92.01 (C-1′), 102.29 (C-5), 116.33 (q, ^1^*J*_CF_ = 291.5 Hz, F_3_CCOO), 143.11 (C-6), 151.30 (C-2), 159.48 (NC(=O)N), 162.97 (q, ^2^*J*_CF_ = 35.4 Hz, F_3_CCOO), 166.10 (C-4), 171.35 (C-7′), 174.73 (C-1‴), 175.35 (C-1^iv^), 176.70 (C-1^v^). ^19^F NMR (282 MHz, D_2_O): δ [ppm] = −75.59 (s, CF_3_). HRMS (ESI^+^): calcd.: 401.2213 [M+2H]^2+^, found: 401.2220. IR (ATR): *ν* = 2962, 1670, 1556, 1464, 1389, 1262, 1199, 1133, 1065, 834, 800, 768. UV (MeCN): λ_max_ = 260. optical rotation: [α]_D_^20^ = +6.7 (c = 0.8, H_2_O). m.p. = 150 °C. Analytical HPLC: *t*_R_ = 6.9 min (method 1). Semipreparative HPLC: *t*_R_ = 16.5 min (method 2).

Uracil*-N-*3-substituted aminoribosylated muraymycin analogue (αβ-**15**) and uracil*-N-*3-substituted non-aminoribosylated muraymycin analogue (**16**): To a solution of the protected uracil*-N-*3-substituted aminoribosylated truncated *N*-Cbz-muraymycin analogue **33** (27.4 mg, 22.0 μmol) in *i*-PrOH (3 mL), 1,4-cyclohexadiene (21.0 μL, 226 μmol) and Pd black (10.0 mg, 94.0 μmol) were added and the mixture was stirred at rt for 1 h. It was then filtered through a syringe filter, and the syringe filter was washed with *i*-PrOH (4 × 5 mL). The solvent of the combined filtrates was evaporated under reduced pressure to give **34** as a colourless wax (24.0 mg) that was directly used in the subsequent peptide coupling step. To a solution of protected urea dipeptide **30** (6.3 mg, 14 μmol) in THF (2.5 mL), HOBt (2.0 mg, 14 μmol), PyBOP (7.4 mg, 14 μmol) and DIPEA (4.8 μL, 28 μmol) were added and the mixture was stirred at rt for 30 min. At 0 °C, a solution of protected uracil*-N-*3–substituted aminoribosylated truncated muraymycin analogue **34** (16.0 mg) in THF (2 mL) was added dropwise, and the reaction mixture was stirred at 0 °C for 1 h and at rt for 3 h. The solvent was evaporated under reduced pressure, and the resultant crude product was purified by column chromatography (97:3, CH_2_Cl_2_-MeOH) to give **35** as a colourless solid (18.4 mg) that was directly used in the subsequent deprotection step. A solution of the protected uracil*-N-*3–substituted aminoribosylated muraymycin analogue **35** (20.0 mg) in TFA (80% in CH_2_Cl_2_, 6.0 mL) was stirred at rt for 30 h. The mixture was then diluted with CH_2_Cl_2_ (20 mL) and the solvent was evaporated under reduced pressure. The resultant residue was dissolved in TFA (10% in water, 5.0 mL), the mixture was stirred at rt for 10 min and then diluted with water (20 mL). The solvent was evaporated under reduced pressure, and the resultant crude product was purified by semipreparative HPLC (method 3) to give αβ-**15** (tris-TFA salt) as a colourless solid (5.1 mg, 1:1 mixture of α,β-anomers, 25% over 3 steps from **33**) and **16** (tris-TFA salt) as a colourless solid (4.5 mg, 27% over 3 steps from **33**). αβ-**15**: ^1^H NMR (500 MHz, D_2_O): δ [ppm] = 0.86 (d, *J* = 6.0 Hz, 3H, 5‴-H_a_), 0.86 (d, *J* = 6.0 Hz, 3H, 5‴-H_a_), 0.92 (d, *J* = 5.9 Hz, 3H, 5‴-H_b_), 0.92 (d, *J* = 5.9 Hz, 3H, 5‴-H_b_), 0.93 (2 d, *J* = 6.9 Hz, 3H, 4^v^-H_a_), 0.93 (2 d, *J* = 6.9 Hz, 3H, 4^v^-H_a_), 0.96 (d, *J* = 6.8 Hz, 3H, 4^v^-H_b_), 0.96 (d, *J* = 6.8 Hz, 3H, 4^v^-H_b_), 1.35–1.51 (m, 2 × 2 H, 2 × 4^iv^-H), 1.52–1.74 (m, 2 × 6 H, 2 × 3‴-H, 2 × 4‴-H, 2 × 3^iv^-H_a_, 2 × 5^iv^-H), 1.74–1.84 (m, 2 × 1 H, 2 × 3^iv^-H_b_), 1.86–1.98 (m, 2 × 4 H, 2 × 2″-H, 2 × 2^vi^-H), 2.16 (dqq, *J* = 6.9, 6.8, 5.4 Hz, 1H, 3^v^-H), 2.16 (dqq, *J* = 6.9, 6.8, 5.4 Hz, 1H, 3^v^-H), 2.32 (ddd, *J* = 15.0, 11.3, 5.7 Hz, 1H, 5′-H_a_), 2.32 (ddd, *J* = 15.0, 11.3, 5.7 Hz, 1H, 5′-H_a_), 2.45 (ddd, *J* = 15.0, 6.4, 2.5 Hz, 1H, 5′-H_b_), 2.45 (ddd, *J* = 15.0, 6.4, 2.5 Hz, 1H, 5′-H_b_), 2.99 (dd, *J* = 7.6, 7.6 Hz, 2H, 6^iv^-H), 2.99 (dd, *J* = 7.6, 7.6 Hz, 2H, 6^iv^-H), 3.04–3.13 (m, 2 × 3H, 2 × 1″-H_b_, 2 × 5^vii^-H_a_), 3.21–3.28 (m, 2 × 1H, 2 × 3″-H_a_), 3.29–3.42 (m, 2 × 2H, 2 × 3″-H_b_, 2 × 5^vii^-H_b_), 3.56 (dt, *J* = 10.2, 6.0, 1H, β-3^vi^-H_a_), 3.63 (dt, *J* = 10.4, 6.2 Hz, 1H, α-3^vi^-H_a_), 3.79–3.87 (m, 2 × 1H, 2 × 3^vi^-H_b_), 3.94 (dd, *J* = 11.3, 6.4 Hz, 1H, 6′-H), 3.94 (dd, *J* = 11.3, 6.4 Hz, 1H, 6′-H), 3.97–4.04 (m, 2 × 3H, 2 × 1^vi^-H, α-3^vii^-H, β-2^vii^-H), 4.04–4.09 (m, 2 × 2H, 2 × 3′-H, 2 × 2^v^-H), 4.09–4.20 (m, 1 × 3H, 1 × 4H, 2 × 4′-H, 2 × 2^iv^-H, α-2^vii^-H, β-3^vii^-H, β-4^vii^-H), 4.20–4.28 (m, 1 × 1H, 1 × 2H, 2 × 2‴-H, α-4^vii^-H), 4.34–4.39 (m, 2 × 1H, 2 × 2′-H), 4.98 (s, 1H, β-1^vii^-H), 5.15 (d, *J* = 4.3 Hz, 1H, α-1^vii^-H), 5.79 (d, *J* = 3.5 Hz, 1H, 1′-H), 5.81 (d, *J* = 3.5 Hz, 1H, 1′-H), 5.94 (d, *J* = 8.1 Hz, 1H, 5-H), 5.94 (d, *J* = 8.1 Hz, 1H, 5-H), 7.64 (d, *J* = 8.1 Hz, 1H, 6-H), 7.64 (d, *J* = 8.1 Hz, 1H, 6-H). ^13^C NMR (126 MHz, D_2_O): δ [ppm] = 16.91 (2 × C_a_-4^v^), 18.43 (2 × C_b_-4^v^), 20.58 (2 × C_a_-5‴), 21.98 (2 × C-4^iv^), 22.04 (2 × C_b_-5‴), 24.32 (2 × C-4‴), 25.56 (2 × C-2″), 26.20 (2 × C-5^iv^), 26.65, 26.89 (α-C-2^vi^_,_ β-C-2^vi^), 29.87 (2 × C-3^v^), 30.70 (2 × C-3^iv^), 32.67 (2 × C-5′), 35.93 (2 × C-3″), 38.96, 39.04 (α-C-1^vi^, β-C-1^vi^), 39.16 (2 × C-6^iv^), 39.42 (2 × C-3‴), 41.37, 43.11 (α-C-5^vii^, β-C-5^vii^), 44.24 (2 × C-1″), 52.51 (2 × C-2‴), 54.02 (2 × C-2^iv^), 58.82 (2 × C-2^v^), 59.32 (2 × C-6′), 66.53 (β-C-3^vi^), 66.67 (α-C-3^vi^), 70.54 (β-C-3^vii^), 70.69 (α-C-3^vii^), 72.52 (α-C-2^vii^) 72.88, 72.92 (2 × C-2′, 2 × C-3′), 74.07 (β-C-2^vii^), 78.07 (β-C-4^vii^), 78.79 (α-C-4^vii^), 79.29, 79,35 (α-C-4′, β-C-4′), 91.79, 92.16 (α-C-1′, β-C-1′), 101.64, 101.68 (α-C-5, β-C-5), 102.31 (α-C-1^vii^), 107.51 (β-C-1^vii^), 116.29 (q, ^1^*J*_CF_ = 291.5 Hz, F_3_CCOO), 116.29 (q, ^1^*J*_CF_ = 291.5 Hz, F_3_CCOO), 140.20, 140.31 (α-C-6, β-C-6), 151.52, 151.57 (α-C-2, β-C-2), 159.46 (2 × NC(=O)N), 162.96 (q, ^2^*J*_CF_ = 35.5 Hz, F_3_CCOO), 162.96 (q, ^2^*J*_CF_ = 35.5 Hz, F_3_CCOO), 165.09, 165.15 (α-C-4, β-C-4), 171.80, 171.85 (α-C-7′, β-C-7′), 174.85 (2 × C-1‴), 175.40 (2 × C-1^iv^), 176.69 (2 × C-1^v^). ^19^F NMR (282 MHz, D_2_O): δ [ppm] = −75.57 (s, CF_3_), –75.57 (s, CF_3_). HRMS (ESI^+^): calcd.: 466.7504 [M+2H]^2+^, found: 466.7507. IR (ATR): *ν* = 3293, 3082, 2961, 1649, 1553, 1462, 1182, 1129, 835, 799, 721. UV (MeCN): λ_max_ = 262. optical rotation: [α]_D_^20^ = +29.3 (c = 0.7, H_2_O). m.p. = 156 °C. Analytical HPLC: *t*_R_ = 6.5 min (method 1). Semipreparative HPLC: *t*_R_ = 14.4 min (method 3). **16**: ^1^H NMR (500 MHz, D_2_O): δ [ppm] = 0.90 (d, *J* = 5.6 Hz, 3H, 5‴-H_a_), 0.94–0.97 (m, 6H, 5‴-H_b_, 4v-H_a_), 0.99 (d, *J* = 6.5 Hz, 3H, 4^v^-H_b_), 1.41–1.53 (m, 2H, 4^iv^-H), 1.55–1.76 (m, 6H, 3‴-H, 4‴-H, 3^iv^-H_a_, 5^iv^-H), 1.78–1.85 (m, 1H, 3^iv^-H_b_), 1.88 (tt, *J* = 7.1, 6.4 Hz, 2H, 2^vi^-H), 1.94 (ddt, *J* = 7.0, 6.9, 6.6 Hz, 2H, 2″-H), 2.19 (dqq, *J* = 6.7, 6.5, 5.9 Hz, 1H, 3^v^-H), 2.31 (ddd, *J* = 15.1, 8.9, 6.3 Hz, 1H, 5′-H_a_), 2.47 (ddd, *J* = 15.1, 5.8, 1.8 Hz, 1H, 5′-H_b_), 3.03 (dd, *J* = 7.5, 7.5 Hz, 2H, 6^iv^-H), 3.07–3.15 (m, 2H, 1″-H), 3.28 (ddd, *J* = 13.3, 6.9, 6.4 Hz, 1H, 3″-H_a_), 3.34 (ddd, *J* = 13.3, 7.0, 6.4 Hz, 1H, 3″-H_b_), 3.67 (t, *J* = 6.4 Hz, 2H, 3^vi^-H), 3.90 (dd, *J* = 6.3, 5.8 Hz, 1H, 6′-H), 4.01 (t, *J* = 7.1 Hz, 2H, 1^vi^-H), 4.08–4.13 (m, 2H, 3′-H, 2^v^-H), 4.15 (dd, *J* = 7.6, 6.0 Hz, 1H, 2^iv^-H), 4.19 (ddd, *J* = 8.9, 7.2, 1.8 Hz, 1H, 4′-H), 4.28 (dd, *J* = 9.3, 4.7 Hz, 1H, 2‴-H), 4.43 (dd, *J* = 3.7, 2.7 Hz, 1H, 2′-H), 5.81 (d, *J* = 2.7 Hz, 1H, 1′-H), 5.97 (d, *J* = 8.1 Hz, 1H, 5-H), 7.67 (d, *J* = 8.1 Hz, 1H, 6-H). ^13^C NMR (126 MHz, D_2_O): δ [ppm] = 16.94 (C_a_-4^v^), 18.48 (C_b_-4^v^), 20.64 (C_a_-5‴), 22.00 (C-4^iv^), 22.07 (C_b_-5‴), 24.36 (C-4‴), 25.62 (C-2″), 26.23 (C-5^iv^), 29.34 (C-2^vi^), 29.94 (C-3^v^), 30.76 (C-3^iv^), 32.87 (C-5′), 35.93 (C-3″), 38.67 (C-1^vi^), 39.20 (C-6^iv^), 39.50 (C-3‴), 44.26 (C-1″), 52.54 (C-2‴), 54.00 (C-2^iv^), 58.91 (C-2^v^), 59.32 (C-3^vi^), 59.93 (C-6′), 72.81 (C-2′), 72.90 (C-3′), 79.80 (C-4′), 92.49 (C-1′), 101.66 (C-5), 116.33 (q, ^1^*J*_CF_ = 291.9 Hz, F_3_CCOO), 140.55 (C-6), 151.55 (C-2), 159.46 (NC(=O)N), 162.99 (q, ^2^*J*_CF_ = 35.5 Hz, F_3_CCOO), 165.19 (C-4), 172.13 (C-7′), 174.85 (C-1‴), 175.35 (C-1^iv^), 176.79 (C-1^v^). ^19^F NMR (282 MHz, D_2_O): δ [ppm] = −75.59 (s, CF_3_). HRMS (ESI^+^): calcd.: 401.2213 [M+2H]^2+^, found: 401.2217. IR (ATR): *ν* = 3306, 2961, 1645, 1554, 1462, 1199, 1181, 1130, 1084, 836, 800, 721. UV (MeCN): λ_max_ = 262. optical rotation: [α]_D_^20^ = +27.0 (c = 0.6, H_2_O). m.p. = 185 °C. Analytical HPLC: *t*_R_ = 6.8 min (method 1). Semipreparative HPLC: *t*_R_ = 16.7 min (method 3).

Protected but-3-enyl β-d-5-azido-5-deoxyriboside (**20**): To a solution of protected β-d-ribosyl fluoride **19** (50.0 mg, 20.0 μmol) [49,50,51,52] in CH_2_Cl_2_ (2 mL) at 0 °C, molecular sieves (4 Å) and a solution of homoallylic alcohol (18.0 mg, 31.0 μmol) in CH_2_Cl_2_ (0.5 mL) were added and the mixture was stirred at 0 °C for 1 h. Boron trifluoride etherate (38 μL, 30 mmol, 0.2 M in CH_2_Cl_2_) was added and the mixture was stirred at 0 °C for further 2 h. It was then quenched with sat. NaHCO_3_ solution, warmed to rt, and filtered. The residue was washed with CH_2_Cl_2_ (3 × 30 mL). The combined organics were washed with brine (100 mL) and dried over Na_2_SO_4_. The solvent was evaporated under reduced pressure, and the resultant crude product was purified by column chromatography (9:1, *iso*-hexane–EtOAc) to give **20** as a colourless oil (40.0 mg, 65%). ^1^H NMR (500 MHz, C_6_D_6_): δ [ppm] = 0.80 (t, *J* = 7.5 Hz, 3H, 1′-H), 0.99 (t, *J* = 7.5 Hz, 3H, 5′-H), 1.42 (q, *J* = 7.5 Hz, 2H, 2′-H), 1.70 (q, *J* = 7.5 Hz, 2H, 4′-H), 2.06–2.17 (m, 2H, 2″-H), 2.70 (dd, *J* = 12.5, 6.4 Hz, 1H, 5-H_a_), 3.02 (dd, *J* = 12.5, 8.1 Hz, 1H, 5-H_b_), 3.12 (dt, *J* = 9.5, 6.6 Hz, 1H, 1″-H_a_), 3.63 (dt, *J* = 9.5, 6.7 Hz, 1H, 1″-H_b_), 4.17 (dd, *J* = 6.0, 0.7 Hz, 1H, 3-H), 4.29 (ddd, *J* = 8.1, 6.4, 0.7 Hz, 1H, 4-H), 4.44 (d, *J* = 6.0 Hz, 1H, 2-H), 4.98–5.04 (m, 2H, 4″-H), 5.08 (s, 1H, 1-H), 5.68 (ddt, *J* = 17.1, 10.3, 6.8 Hz, 1H, 3″-H). ^13^C NMR (126 MHz, C_6_D_6_): δ [ppm] = 7.47 (C-5′), 8.29 (C-1′), 29.13 (C-4′), 29.41 (C-2′), 34.03 (C-2″), 53.57 (C-5), 67.03 (C-1″), 82.52 (C-3), 85.62 (C-2), 85.74 (C-4), 109.00 (C-1), 116.43 (C-4″), 116.63 (C-3′), 135.06 (C-3″). HRMS (ESI^+^): calcd.: 320.1581 [M+Na]^+^, found: 320.1525. IR (ATR): *ν* = 2974, 2939, 2882, 2100, 1464, 1360, 1273, 926, 847. optical rotation: [α]_D_^20^ = −47.5 (c = 1.7, CHCl_3_). TLC: R*_f_* = 0.49 (9:1, *iso*-hexane–Et_2_O).

Protected but-3-enyl β-d-5-amino-5-deoxyriboside (**21**): To a solution of protected but-3-enyl β-d-5-azido-5-deoxyriboside **20** (123 mg, 0.414 mmol) in THF/toluene (1:1, 6 mL), PPh_3_ (326 mg, 1.24 mmol) and water (373 μL, 20.7 mmol) were added and the mixture was stirred at 50 °C for 13 h. After cooling to rt, di–*tert*–butyldicarbonate (181 mg, 0.827 mmol) and NaHCO_3_ (70.0 mg, 0.830 mmol) were added and the mixture was stirred at rt for 1.5 h. It was then diluted with EtOAc (150 mL), washed with water (150 mL) and brine (150 mL) and dried over Na_2_SO_4_. The solvent was evaporated under reduced pressure, and the resultant crude product was purified by column chromatography (9:1, *iso*-hexane–EtOAc) to give **21** as a colourless oil (150 mg, 98%). ^1^H NMR (500 MHz, C_6_D_6_): δ [ppm] = 0.78 (t, *J* = 7.5 Hz, 3H, 1′-H), 0.99 (t, *J* = 7.4 Hz, 3H, 5′-H), 1.41 (q, *J* = 7.5 Hz, 2H, 2′-H), 1.43 (s, 9H, OC(CH_3_)_3_), 1.69 (q, *J* = 7.4 Hz, 2H, 4′-H), 2.08–2.20 (m, 2H, 2″-H), 3.11 (dt, *J* = 9.4, 6.5 Hz, 1H, 1″-H_a_), 3.20–3.30 (m, 2H, 5-H), 3.53 (dt, *J* = 9.4, 6.7 Hz, 1H, 1″-H_b_), 4.31 (dd, *J* = 6.0, 6.0 Hz, 1H, 4-H), 4.46 (d, *J* = 5.9 Hz, 1H, 3-H), 4.53 (d, *J* = 5.9 Hz, 1H, 2-H), 4.96 (s, 1H, NH), 4.98–5.05 (m, 2H, 4″-H), 5.08 (s, 1H, 1-H), 5.70 (ddt, *J* = 17.1, 10.3, 6.8 Hz, 1H, 3″-H). ^13^C NMR (126 MHz, C_6_D_6_): δ [ppm] = 7.75 (C-5′), 8.60 (C-1′), 28.49 (OC(CH_3_)_3_), 29.38 (C-4′), 29.62 (C-2′), 34.30 (C-2″), 44.18 (C-5), 67.43 (C-1″), 78.88 (OC(CH_3_)_3_), 82.79 (C-3), 86.45 (C-2), 86.76 (C-4), 109.32 (C-1), 116.58 (C-3′), 116.79 (C-4″), 135.06 (C-3″), 156.01 (Boc-C=O). HRMS (ESI^+^): calcd.: 394.2200 [M+Na]^+^, found: 394.2213. IR (ATR): *ν* = 2974, 1716, 1699, 1514, 1365, 1248, 1167, 924, 872. optical rotation: [α]_D_^20^ = −30.5 (c = 1.9, CHCl_3_). TLC: R*_f_* = 0.15 (9:1, *iso*-hexane–Et_2_O).

Protected 3-oxopropyl β-d-5-amino-5-deoxyriboside (**22**): Ozone was bubbled through a solution of protected but-3-enyl β-d-5-amino-5-deoxyriboside **21** (106 mg, 0.269 mmol) in CH_2_Cl_2_ (3 mL), MeOH (26 mL) and pyridine (87 μL, 1.1 mmol) at −78 °C for 15 min. Nitrogen was then bubble through the solution at −78 °C for 45 min, and dimethyl sulfide (198 μL, 2.69 mmol) was added. The reaction mixture was stirred overnight and slowly warmed to rt during this period. The solvent was evaporated under reduced pressure, and the resultant crude product was purified by column chromatography (7:3, *iso*-hexane–EtOAc) to give **22** as a colourless oil (87.0 mg, 86%). ^1^H NMR (500 MHz, C_6_D_6_, 70 °C): δ [ppm] = 0.79 (t, *J* = 7.4 Hz, 3H, 1′-H), 0.96 (t, *J* = 7.4 Hz, 3H, 5′-H), 1.40–1.48 (m, 2H, 2′-H), 1.43 (s, 9H, OC(CH_3_)_3_), 1.68 (q, *J* = 7.4 Hz, 2H, 4′-H), 2.01–2.13 (m, 2H, 2″-H), 3.12–3.19 (m, 2H, 5-H), 3.23 (ddd, *J* = 10.1, 6.6, 5.3 Hz, 1H, 1″-H_a_), 3.68 (ddd, *J* = 10.1, 6.8, 5.4 Hz 1H, 1″-H_b_), 4.29 (dd, *J* = 6.4, 6.3 Hz, 1H, 4-H), 4.42 (d, *J* = 6.1 Hz, 1H, 3-H), 4.45 (d, *J* = 6.1 Hz, 1H, 2-H), 4.84 (s, 1H, NH), 4.99 (s, 1H, 1-H), 9.33 (dd, *J* = 1.6, 1.5 Hz, 1H, 3″-H). ^13^C NMR (126 MHz, C_6_D_6_, 70 °C): δ [ppm] = 7.19 (C-5′), 7.89 (C-1′), 28.06 (OC(CH_3_)_3_), 29.21 (C-4′), 29.33 (C-2′), 43.20 (C-2″), 44.04 (C-5), 61.30 (C-1″), 78.66 (OC(CH_3_)_3_), 82.47 (C-3), 86.01 (C-2), 86.45 (C-4), 109.15 (C-1), 116.42 (C-3′), 155.61 (Boc-C=O), 198.24 (C-3″). HRMS (ESI^+^): calcd.: 396.1993 [M+Na]^+^, found: 396.2005. IR (ATR): *ν* = 1713, 1695, 1518, 1365, 1257, 1167, 1011, 924, 791. optical rotation: [α]_D_^20^ = −17.6 (c = 1.7, CHCl_3_). TLC: R*_f_* = 0.15 (7:3, *iso*-hexane–EtOAc).

Protected 3-hydroxypropyl β-d-5-amino-5-deoxyriboside (**23**): Ozone was bubbled through a solution of protected but-3-enyl β-d-5-amino-5-deoxyriboside **21** (70.0 mg, 19.0 μmol) in CH_2_Cl_2_ (3 mL), MeOH (26 mL) and pyridine (61 μL, 0.75 mmol) at −78 °C for 30 min. Dimethyl sulfide (138 μL, 1.88 mmol) was then added. The reaction mixture was stirred overnight and slowly warmed to rt during this period. The solvent was evaporated under reduced pressure, and the resultant crude aldehyde **22** was dissolved in MeOH (10 mL). At 0 °C, sodium borohydride (34.0 mg, 1.90 mmol) was added and the mixture was stirred at rt for 30 min. After addition of sat. NH_4_Cl solution, the aqueous layer was extracted with EtOAc (3 × 30 mL). The combined organics were washed with water (2 × 20 mL) and dried over Na_2_SO_4_. The solvent was evaporated under reduced pressure, and the resultant crude product was purified by column chromatography (65:35, *iso*-hexane-EtOAc) to give **23** as a colourless oil (66.0 mg, 95%). ^1^H NMR (500 MHz, C_6_D_6_): δ [ppm] = 0.80 (t, *J* = 7.5 Hz, 3H, 1′-H), 0.99 (t, *J* = 7.5 Hz, 3H, 5′-H), 1.38–1.47 (m, 2H, 2′-H), 1.43 (s, 9H, OC(CH_3_)_3_), 1.51–1.59 (m, 2H, 2″-H), 1.69 (q, *J* = 7.4 Hz, 2H, 4′-H), 3.14 (ddd, *J* = 9.6, 5.5, 5.5 Hz, 1H, 1″-H_a_), 3.19–3.26 (m, 2H, 5-H), 3.44–3.51 (m, 1H, 3″-H_a_), 3.51–3.58 (m, 1H, 3″-H_b_), 3.72–3.79 (m, 1H, 1″-H_b_), 4.32 (dd, *J* = 6.6, 5.9 Hz, 1H, 4-H), 4.41 (d, *J* = 6.0 Hz, 1H, 3-H), 4.50 (d, *J* = 6.0 Hz, 1H, 2-H), 5.05 (s, 1H, NH), 5.10 (s, 1H, 1-H). ^13^C NMR (126 MHz, C_6_D_6_): δ [ppm] = 7.76 (C-5′), 8.60 (C-1′), 28.48 (OC(CH_3_)_3_), 29.39 (C-4′), 29.63 (C-2′), 32.60 (C-2″), 44.42 (C-5), 59.43 (C-3″), 64.87 (C-1″), 79.09 (OC(CH_3_)_3_), 82.76 (C-3), 86.42 (C-2), 86.65 (C-4), 109.38 (C-1), 116.64 (C-3′), 156.23 (Boc-C=O). HRMS (ESI^+^): calcd.: 398.2149 [M+Na]^+^, found: 398.2130. IR (ATR): *ν* = 2936, 1689, 1365, 1250, 1167, 1088, 1012, 972, 924. optical rotation: [α]_D_^20^ = −30.2 (c = 1.7, CHCl_3_). TLC: R*_f_* = 0.12 (7:3, *iso*-hexane–EtOAc).

Protected 3-(tosyloxy)propyl β-d-5-amino-5-deoxyriboside (**24**): To a solution of protected 3-hydroxypropyl β-d-5-amino-5-deoxyriboside **23** (77.0 mg, 0.205 mmol) in CH_2_Cl_2_ (5 mL) at 0 °C, pyridine (38.0 μL, 0.472 mmol) and tosyl chloride (50.8 mg, 0.267 mmol) were added. The mixture was slowly warmed to rt and then stirred at rt for 3 d. Water (20 mL) was added, and the aqueous layer was extracted with CH_2_Cl_2_ (2 × 30 mL). The combined organics were washed with brine (30 mL) and dried over Na_2_SO_4_. The solvent was evaporated under reduced pressure, and the resultant crude product was purified by column chromatography (4:1, *iso*-hexane–EtOAc) to give **24** as a colourless oil (86.0 mg, 79%). ^1^H NMR (500 MHz, C_6_D_6_): δ [ppm] = 0.78 (t, *J* = 7.5 Hz, 3H, 1′-H), 0.97 (t, *J* = 7.5 Hz, 3H, 5′-H), 1.41 (q, *J* = 7.5 Hz, 2H, 2′-H), 1.43 (s, 9H, OC(CH_3_)_3_), 1.44–1.52 (m, 2H, 2″-H), 1.67 (q, *J* = 7.5 Hz, 2H, 4′-H), 1.88 (s, 3H, aryl–CH_3_), 2.92 (ddd, *J* = 9.8, 6.3, 6.3 Hz, 1H, 1″-H_a_), 3.02–3.10 (m, 1H, 3″-H_a_), 3.12–3.20 (m, 1H, 3″-H_b_), 3.37 (ddd, *J* = 9.8, 5.7, 5.7 Hz, 1H, 1″-H_b_), 3.90–3.99 (m, 2H, 5-H), 4.22 (dd, *J* = 6.3, 6.3 Hz, 1H, 4-H), 4.33 (d, *J* = 6.0 Hz, 1H, 3-H), 4.38 (d, *J* = 6.0 Hz, 1H, 2-H), 4.67 (dd, *J* = 5.4, 5.4 Hz, 1H, NH), 4.85 (s, 1H, 1-H), 6.77 (d, *J* = 8.2 Hz, 2H, 3‴-H, 5‴-H), 7.77 (d, *J* = 8.2 Hz, 2H, 2‴-H, 6‴-H). ^13^C NMR (126 MHz, C_6_D_6_): δ [ppm] = 7.74 (C-5′), 8.60 (C-1′), 21.18 (aryl–CH_3_), 28.46 (OC(CH_3_)_3_), 29.26 (C-2″), 29.38 (C-4′), 29.65 (C-2′), 44.14 (C-5), 63.25 (C-3″), 67.13 (C-1″), 79.04 (OC(CH_3_)_3_), 82.70 (C-3), 86.20 (C-2), 86.75 (C-4), 109.05 (C-1), 116.61 (C-3′), 128.20 (C-2‴, C-6‴), 129.87 (C-3‴, C-5‴), 134.49 (C-4‴), 144.24 (C-1‴), 155.94 (NC(=O)O). HRMS (ESI^+^): calcd.: 552.2238 [M+Na]^+^, found: 552.2237. IR (ATR): *ν* = 1713, 1364, 1174, 1095, 924, 837, 814, 752, 663. UV (MeCN): λ_max_ = 225, 262, 273. optical rotation: [α]_D_^20^ = −22.8 (c = 2.0, CHCl_3_). TLC: R*_f_* = 0.18 (4:1, *iso*-hexane–EtOAc).

Protected 6′*-N-*substituted aminoribosylated (6′*S*)-nucleosyl amino acid (**26**): To a solution of the protected (6′*S*)-nucleosyl amino acid **25** (83.0 mg, 0.142 mmol) [35,36] in THF (6 mL) over molecular sieves (4 Å), aldehyde **22** (63.0 mg, 0.169 mmol) was added and the mixture was stirred at rt for 24 h. Amberlyst™ 15 (6.6 mg, 31 μmol) and sodium triacetoxyborohydride (60.0 mg, 0.284 mmol) were added, and the mixture was stirred at rt for 22 h. It was then filtered, the filtrate was diluted with EtOAc (100 mL) and washed with sat. Na_2_CO_3_ solution (100 mL). The aqueous layer was extracted with EtOAc (3 × 50 mL), and the combined organics were dried over Na_2_SO_4_. The solvent was evaporated under reduced pressure, and the resultant crude product was purified by column chromatography (97.5:2.5, CH_2_Cl_2_-MeOH) to give **26** as a colourless solid (101 mg, 76%). ^1^H NMR (500 MHz, CD_3_OD): δ [ppm] = 0.09 (s, 3H, SiCH_3_), 0.10 (s, 3H, SiCH_3_), 0.12 (s, 3H, SiCH_3_), 0.13 (s, 3H, SiCH_3_), 0.87 (t, *J* = 7.5 Hz, 3H, 1^iv^-H), 0.89 (t, *J* = 7.5 Hz, 3H, 5^iv^-H), 0.91 (s, 9H, SiC(CH_3_)_3_), 0.93 (s, 9H, SiC(CH_3_)_3_), 1.44 (s, 9H, NC(=O)OC(CH_3_)_3_), 1.49 (s, 9H, C(=O)OC(CH_3_)_3_), 1.58 (q, *J* = 7.5 Hz, 2H, 2^iv^-H), 1.67 (q, *J* = 7.5 Hz, 2H, 4^iv^-H), 1.70–1.82 (m, 2H, 2″-H), 1.86–1.94 (m, 1H, 5′-H_a_), 2.00–2.08 (m, 1H, 5′-H_b_), 2.58–2.65 (m, 1H, 1″-H_a_), 2.65–2.72 (m, 1H, 1″-H_b_), 3.17 (d, *J* = 7.2 Hz, 2H, 5‴-H), 3.34 (dd, *J* = 9.2, 4.7 Hz, 1H, 6′-H), 3.41–3.47 (m, 1H, 3″-H_a_), 3.74–3.80 (m, 1H, 3″-H_b_), 3.91 (dd, *J* = 4.6, 4.4 Hz, 1H, 3′-H), 4.04–4.10 (m, 1H, 4′-H), 4.16 (dd, *J* = 7.2, 7.2 Hz, 1H, 4‴-H), 4.33 (dd, *J* = 4.4, 4.1 Hz, 1H, 2′-H), 4.60 (d, *J* = 6.0 Hz, 1H, 3‴-H), 4.67 (d, *J* = 6.0 Hz, 1H, 2‴-H), 5.01 (s, 1H, 1‴-H), 5.75 (d, *J* = 8.1 Hz, 1H, 5-H), 5.78 (d, *J* = 4.1 Hz, 1H, 1′-H), 7.65 (d, *J* = 8.1 Hz, 1H, 6-H). ^13^C NMR (126 MHz, CD_3_OD): δ [ppm] = −4.40 (SiCH_3_), –4.39 (SiCH_3_), –4.39 (SiCH_3_), −3.98 (SiCH_3_), 7.75 (C-5^iv^), 8.72 (C-1^iv^), 18.88 (SiC(CH_3_)_3_), 18.94 (SiC(CH_3_)_3_, 26.40 (SiC(CH_3_)_3_), 26.47 (SiC(CH_3_)_3_), 28.47 (NC(=O)OC(CH_3_)_3_), 28.79 (C(=O)OC(CH_3_)_3_), 29.92 (C-4^iv^), 30.31 (C-2^iv^), 30.54 (C-2″), 38.00 (C-5′), 44.63 (C-5‴), 46.10 (C-1″), 60.70 (C-6′), 67.13 (C-3″), 75.92 (C-2′), 76.60 (C-3′), 80.28 (NC(=O)OC(CH_3_)_3_), 82.57 (C(=O)OC(CH_3_)_3_), 82.92 (C-4′), 83.83 (C-3‴), 87.09 (C-2‴), 87.20 (C-4‴), 91.87 (C-1′), 103.04 (C-5), 110.00 (C-1‴), 117.68 (C-3^iv^), 142.68 (C-6), 152.15 (C-2), 158.31 (Boc-C=O), 166.01 (C-4), 174.84 (C-7′). HRMS (ESI^+^): calcd.: 943.5490 [M+H]^+^, found: 943.5486. IR (ATR): *ν* = 2931, 2858, 1692, 1561, 1366, 1251, 1157, 1090, 836, 776. UV (MeCN): λ_max_ = 261. optical rotation: [α]_D_^20^ = −15.0 (c = 1.0, CHCl_3_). m.p. = 62 °C. TLC: R*_f_* = 0.30 (94:6, CH_2_Cl_2_-MeOH).

Protected 6′*-N-*substituted aminoribosylated truncated *N*-Cbz-muraymycin analogue (**28**): To a solution of the protected 6′*-N-*substituted aminoribosylated (6′*S*)–nucleosyl amino acid **26** (88.0 mg, 93.3 μmol) in THF (6 mL) over molecular sieves (4 Å), aldehyde **27** (33.0 mg, 103 μmol) [20] was added and the mixture was stirred at rt for 39 h. Amberlyst™ 15 (4.3 mg, 21 μmol) and sodium triacetoxyborohydride (39.3 mg, 187 μmol) were added, and the mixture was stirred at rt for 15 h. More aldehyde **27** (33.0 mg, 103 μmol) and, after 4 h, more sodium triacetoxyborohydride (39.3 mg, 187 μmol) was added. The mixture was stirred at rt for 18 h, then more sodium triacetoxyborohydride (39.3 mg, 187 μmol) was added and stirring at rt was continued for 2 h. The mixture was then filtered, the filtrate was diluted with EtOAc (100 mL) and washed with sat. Na_2_CO_3_ solution (70 mL). The aqueous layer was extracted with EtOAc (3 × 50 mL), and the combined organics were dried over Na_2_SO_4_. The solvent was evaporated under reduced pressure, and the resultant crude product was purified by column chromatography (1. 98.2:1.8→98:2, CH_2_Cl_2_-MeOH, 2. 98:2, CH_2_Cl_2_-MeOH) to give **28** as a colourless solid (81.6 mg, 70%). ^1^H NMR (500 MHz, CD_3_OD): δ [ppm] = 0.06 (s, 3H, SiCH_3_), 0.10 (s, 3H, SiCH_3_), 0.13 (s, 3H, SiCH_3_), 0.15 (s, 3H, SiCH_3_), 0.86 (t, *J* = 7.5 Hz, 3H, 1^vii^-H), 0.88–0.93 (m, 3H, 5^vii^-H), 0.90 (s, 9H, SiC(CH_3_)_3_), 0.94 (s, 9H, SiC(CH_3_)_3_), 0.92–0.97 (m, 6H, 5‴-H), 1.44 (s, 9H, NC(=O)OC(CH_3_)_3_), 1.48 (s, 9H, C(=O)OC(CH_3_)_3_), 1.51–1.57 (m, 2H, 3‴-H), 1.57 (q, *J* = 7.5 Hz, 2H, 2^vii^-H), 1.67 (q, *J* = 7.5 Hz, 2H, 4^vii^-H), 1.62–1.75 (m, 5H, 2″-H, 4‴-H, 2^v^-H), 1.77–1.85 (m, 1H, 5′-H_a_), 2.17–2.27 (m, 1H, 5′-H_b_), 2.52–2.62 (m, 2H, 1″-H_a,_ 1^v^-H_a_), 2.64–2.67 (m, 2H, 1″-H_b_, 1^v^-H_b_), 3.13–3.19 (m, 2H, 5^vi^-H), 3.22 (dd, *J* = 6.7, 6.7 Hz, 2H, 3″-H), 3.40–3.47 (m, 1H, 3^v^-H_a_), 3.50 (dd, *J* = 10.6, 3.5 Hz, 1H, 6′-H), 3.66–3.73 (m, 1H, 3^v^-H_b_), 3.94 (dd, *J* = 4.5, 4.1 Hz, 1H, 3′-H), 3.96–4.02 (m, 1H, 4′-H), 4.12 (dd, *J* = 8.9, 7.7 Hz, 1H, 2‴-H), 4.15 (dd, *J* = 7.3, 6.8 Hz, 1H, 4^vi^-H), 4.40 (dd, *J* = 4.8, 4.5 Hz, 1H, 2′-H), 4.59 (d, *J* = 6.0 Hz, 1H, 3^vi^-H), 4.67 (d, *J* = 6.0 Hz, 1H, 2^vi^-H), 5.01 (s, 1H, 1^vi^-H), 5.07 (d, *J* = 12.5 Hz, 1H, 1^iv^-H_a_), 5.11 (d, *J* = 12.5 Hz, 1H, 1^iv^-H_b_), 5.76 (d, *J* = 8.1 Hz, 1H, 5-H), 5.78 (d, *J* = 4.8 Hz, 1H, 1′-H), 7.24–7.38 (m, 5H, 3^iv^-H, 4^iv^-H, 5^iv^-H, 6^iv^-H, 7^iv^-H,), 7.61 (d, *J* = 8.1 Hz, 1H, 6-H). ^13^C NMR (126 MHz, CD_3_OD): δ [ppm] = −4.43 (SiCH_3_), –4.31 (SiCH_3_), –4.23 (SiCH_3_), −3.98 (SiCH_3_), 7.80 (C-5^vii^), 8.74 (C-1^vii^), 18.86 (SiC(CH_3_)_3_), 19.00 (SiC(CH_3_)_3_), 22.06 (C_a_-5‴), 23.48 (C_b_-5‴), 25.95 (C-4‴), 26.39 (SiC(CH_3_)_3_), 26.49 (SiC(CH_3_)_3_), 28.68 (C(=O)OC(CH_3_)_3_), 28.81 (NC(=O)OC(CH_3_)_3_), 29.26 (C-2″), 29.93 (C-4^vii^), 30.13 (C-2^v^), 30.33 (C-2^vii^), 35.38 (C-5′), 38.59 (C-3″), 42.36 (C-3‴), 44.67 (C-5^vi^), 49.28 (C-1^v^), 49.97 (C-1″), 55.12 (C-2‴), 61.91 (C-6′), 66.82 (C-3^v^), 67.73 (C-1^iv^), 75.63 (C-2′), 76.96 (C-3′), 80.27 (NC(=O)OC(CH_3_)_3_), 82.60 (C(=O)OC(CH_3_)_3_), 83.35 (C-4′), 83.88 (C-2^vi^), 87.03 (C-3^vi^), 87.10 (C-4^vi^), 91.76 (C-1′), 103.23 (C-5), 110.00 (C-1^vi^), 117.67 (C-3^vii^), 128.88, 129.04, 129.49 (C-3^iv^, C-4^iv^, C-5^iv^, C-6^iv^ C-7^iv^), 138.18 (C-2^iv^), 142.84 (C-6), 152.15 (C-2), 158.28, 158.34 (Cbz–C=O, Boc-C=O), 165.94 (C-4), 172.89, 175.19 (C-7′, C-1‴). HRMS (ESI^+^): calcd.: 1247.7277 [M+H]^+^, found: 1247.7252. IR (ATR): *ν* = 2929, 2857, 1692, 1366, 1258, 1153, 1087, 1043, 836, 777. UV (MeCN): λ_max_ = 260. optical rotation: [α]_D_^20^ = −5.0 (c = 1.0, CHCl_3_). m.p. = 75 °C. TLC: R*_f_* = 0.26 (96:4, CH_2_Cl_2_-MeOH).

Protected 6′*-N-*substituted aminoribosylated truncated muraymycin analogue (**29**): To a solution of the protected 6′*-N-*substituted aminoribosylated truncated *N*-Cbz–muraymycin analogue **28** (28.2 mg, 22.6 μmol) in *i*-PrOH (3 mL), 1,4–cyclohexadiene (21.0 μL, 226 μmol) and Pd black (10.0 mg, 94.0 μmol) were added and the mixture was stirred at rt for 1.5 h. It was then filtered through a syringe filter, and the syringe filter was washed with *i*-PrOH (4 × 5 mL). The solvent of the combined filtrates was evaporated under reduced pressure to give **29** as a colourless wax (25.2 mg, quant.). ^1^H NMR (500 MHz, CD_3_OD): δ [ppm] = 0.07 (s, 3 H, SiCH_3_), 0.10 (s, 3H, SiCH_3_), 0.13 (s, 3H, SiCH_3_), 0.15 (s, 3H, SiCH_3_), 0.87 (t, *J* = 7.5 Hz, 3H, 1^vi^-H), 0.89 (t, *J* = 7.5 Hz, 3H, 5^vi^-H), 0.90 (s, 9H, SiC(CH_3_)_3_), 0.95 (s, 9H, SiC(CH_3_)_3_), 0.95–1.02 (m, 6H, 5‴-H), 1.45 (s, 9H, NC(=O)OC(CH_3_)_3_), 1.49 (s, 9H, C(=O)OC(CH_3_)_3_), 1.58 (q, *J* = 7.5 Hz, 2H, 2^vi^-H), 1.67 (q, *J* = 7.5 Hz, 2H, 4^vi^-H), 1.63–1.77 (m, 7H, 2″-H, 3‴-H, 4‴-H, 2^iv^-H), 1.77–1.85 (m, 1H, 5′-H_a_), 2.17–2.26 (m, 1H, 5′-H_b_), 2.56–2.65 (m, 2H, 1″-H_a,_ 1^iv^-H_a_), 2.66–2.78 (m, 2H, 1″-H_b_, 1^iv^-H_b_), 3.13–3.26 (m, 3H, 3″-H_a_, 5^v^-H), 3.33–3.41 (m, 1H, 3″-H_b_), 3.42–3.49 (m, 1H, 3^iv^-H_a_), 3.51 (dd, *J* = 10.5, 3.5 Hz, 1H, 6′-H), 3.68–3.75 (m, 1H, 3^iv^-H_b_), 3.76–3.81 (m, 1H, 2‴-H), 3.95 (dd, *J* = 4.4, 4.1 Hz, 1H, 3′-H), 3.96–4.01 (m, 1H, 4′-H), 4.13–4.19 (m, 1H, 4^v^-H), 4.43 (dd, *J* = 4.8, 4.4 Hz, 1H, 2′-H), 4.58–4.62 (m, 1H, 3^v^-H), 4.68 (d, *J* = 6.0 Hz, 1H, 2^v^-H), 4.99–5.02 (m, 1H, 1^v^-H), 5.76 (d, *J* = 4.8 Hz, 1H, 1′-H), 5.77 (d, *J* = 8.0 Hz, 1H, 5-H), 7.60 (d, *J* = 8.0 Hz, 1H, 6-H). ^13^C NMR (126 MHz, CD_3_OD): δ [ppm] = −4.43 (SiCH_3_), –4.34 (SiCH_3_), –4.27 (SiCH_3_), –3.98 (SiCH_3_), 7.79 (C-5^vi^), 8.73 (C-1^vi^), 18.86 (SiC(CH_3_)_3_), 19.00 (SiC(CH_3_)_3_), 22.29 (C_a_-5‴), 23.08 (C_b_-5‴), 25.56 (C-4‴), 26.38 (SiC(CH_3_)_3_), 26.48 (SiC(CH_3_)_3_), 28.67 (C(=O)OC(CH_3_)_3_), 28.81 (NC(=O)OC(CH_3_)_3_), 29.42 (C-2″), 29.93 (C-4^vi^), 30.06 (C-2^iv^), 30.32 (C-2^vi^), 35.10 (C-5′), 39.07 (C-3″), 41.99 (C-3‴), 44.63 (C-5^v^), 50.05 (C-1^iv^), 50.27 (C-1″), 53.25 (C-2‴), 62.04 (C-6′), 66.83 (C-3^iv^), 75.61 (C-2′), 76.99 (C-3′), 80.30 (NC(=O)OC(CH_3_)_3_), 82.69 (C(=O)OC(CH_3_)_3_), 83.29 (C-4′), 83.89 (C-2^v^), 87.04 (C-3^v^), 87.12 (C-4^v^), 92.13 (C-1′), 103.15 (C-5), 109.91 (C-1^v^), 117.71 (C-3^vi^), 142.95 (C-6), 152.09 (C-2), 158.32 (Boc-C=O), 166.04 (C-4), 170.68, 172.93 (C-7′, C-1‴). HRMS (ESI^+^): calcd.: 1113.6897 [M+H]^+^, found: 1113.6909. IR (ATR): *ν* = 2961, 2929, 1695, 1366, 1258, 1149, 1085, 1014, 867, 797. UV (MeCN): λ_max_ = 260. optical rotation: [α]_D_^20^ = −53.8 (c = 1.3, CHCl_3_). TLC: R*_f_* = 0.10 (95:5, CH_2_Cl_2_-MeOH).

Protected uracil*-N-*3-substituted aminoribosylated truncated *N*-Cbz-muraymycin analogue (**33**): To a solution of protected truncated muraymycin analogue **32** (81.0 mg, 91.0 μmol) [36] in MeCN (10 mL), tosylate **24** (48.2 mg, 91.0 μmol) and K_2_CO_3_ (13.8 mg, 100 μmol) were added. Over a period of 8 h, the mixture was stirred and slowly heated to reflux, and then it was stirred under reflux for 16 h. After cooling to rt, it was diluted with EtOAc (150 mL) and washed with water (100 mL). The organic layer was dried over Na_2_SO_4_, and the solvent was evaporated under reduced pressure. The resultant crude product was purified by column chromatography (98:2, CH_2_Cl_2_-MeOH) to give **33** as a colourless solid (79.1 mg, 70%). ^1^H NMR (500 MHz, CD_3_OD): δ [ppm] = 0.11 (s, 3H, SiCH_3_), 0.11 (s, 3H, SiCH_3_), 0.11 (s, 3H, SiCH_3_), 0.13 (s, 3H, SiCH_3_), 0.87–0.92 (m, 3H, 5^vii^-H), 0.88 (t, *J* = 7.5 Hz, 3H, 1^vii^-H), 0.89–0.96 (m, 6H, 5‴-H), 0.91 (s, 9H, SiC(CH_3_)_3_), 0.93 (s, 9H, SiC(CH_3_)_3_), 1.44 (s, 9H, NC(=O)OC(CH_3_)_3_), 1.49 (s, 9H, C(=O)OC(CH_3_)_3_), 1.51–1.56 (m, 2H, 3‴-H), 1.58 (q, *J* = 7.5 Hz, 2H, 2^vii^-H), 1.64–1.72 (m, 3H, 2″-H, 4‴-H), 1.67 (q, *J* = 7.5 Hz, 2H, 4^vii^-H), 1.83–1.90 (m, 2H, 2^v^-H), 1.88–1.96 (m, 1H, 5′-H_a_), 2.02–2.10 (m, 1H, 5′-H_b_), 2.52–2.59 (m, 1H, 1″-H_a_), 2.60–2.68 (m, 1H, 1″-H_b_), 3.13–3.18 (m, 2H, 5^vi^-H), 3.21–3.29 (m, 2H, 3″-H), 3.25 (dd, *J* = 9.7, 4.3 Hz, 1H, 6′-H), 3.39–3.46 (m, 1H, 3^v^-H_a_), 3.72–3.78 (m, 1H, 3^v^-H_b_), 3.85 (dd, *J* = 5.3, 4.3 Hz, 1H, 3′-H), 4.00 (dd, *J* = 6.9, 6.9 Hz, 2H, 1^v^-H) 4.07–4.13 (m, 2H, 4′-H, 2‴-H), 4.16 (dd, *J* = 7.1, 7.1 Hz, 1H, 4^vi^-H), 4.31 (dd, *J* = 4.3, 3.6 Hz, 1H, 2′-H), 4.55 (d, *J* = 6.0 Hz, 1H, 3^vi^-H), 4.66 (d, *J* = 6.0 Hz, 1H, 2^vi^-H), 5.00 (s, 1H, 1^vi^-H), 5.05–5.12 (m, 2H, 1^iv^-H), 5.80 (d, *J* = 3.6 Hz, 1H, 1′-H), 5.84 (d, *J* = 8.0 Hz, 1H, 5-H), 7.25–7.38 (m, 5H, C-3^iv^, C-4^iv^, C-5^iv^, C-6^iv^, C-7^iv^), 7.64 (d, *J* = 8.1 Hz, 1H, 6-H). ^13^C NMR (126 MHz, CD_3_OD): δ [ppm] = −4.47 (SiCH_3_), –4.41 (SiCH_3_), –4.17 (SiCH_3_), –3.93 (SiCH_3_), 7.77 (C-5^vii^), 8.81 (C-1^vii^), 18.91 (SiC(CH_3_)_3_), 18.93 (SiC(CH_3_)_3_), 22.01 (C_a_-5‴), 23.44 (C_b_-5‴), 25.94 (C-4‴), 26.47 (SiC(CH_3_)_3_), 26.48 (SiC(CH_3_)_3_), 28.47 (C(=O)OC(CH_3_)_3_), 28.67 (C-2^v^), 28.78 (NC(=O)OC(CH_3_)_3_), 29.91 (C-4^vii^), 30.35 (C-2^vii^), 30.73 (C-2″), 37.95 (C-5′), 38.20 (C-3″), 40.04 (C-1^v^), 42.28 (C-3‴), 44.62 (C-5^vi^), 46.09 (C-1″), 55.23 (C-2‴), 60.75 (C-6′), 67.11 (C-3^v^), 67.72 (C-1^iv^), 75.21 (C-2′), 76.52 (C-3′), 80.27 (NC(=O)OC(CH_3_)_3_), 82.24 (C-4′), 83.03 (C(=O)OC(CH_3_)_3_), 83.84 (C-2^vi^), 87.05 (C-3^vi^), 87.15 (C-4^vi^), 92.75 (C-1′), 102.41 (C-5), 110.14 (C-1^vi^), 117.64 (C-3^vii^), 128.88, 129.04, 129.48 (C-3^iv^, C-4^iv^, C-5^iv^, C-6^iv^, C-7^iv^), 138.18 (C-2^iv^), 140.52 (C-6), 152.33 (C-2), 158.32, 158.35 (Boc-C=O, Cbz–C=O), 164.83 (C-4), 174.56, 175.49 (C-7′, C-1‴). HRMS (ESI^+^): calcd.: 1247.7277 [M+H]^+^, found: 1247.7292. IR (ATR): *ν* = 2959, 2930, 2857, 1708, 1663, 1258, 1156, 1091, 802, 778. UV (MeCN): λ_max_ = 262. optical rotation: [α]_D_^20^ = −4.5 (c = 1.1, CHCl_3_). m.p. = 66 °C. TLC: R*_f_* = 0.13 (96:4, CH_2_Cl_2_-MeOH).

### 4.2. Overexpression of MraY from S. aureus

The overexpression of MraY was performed as described before [33,34,43].

### 4.3. Fluorescence-Based MraY Assay

The assay was performed as described before [33,34,43].

## 5. Conclusions

In summary, we have successfully synthesised chemically tractable aminoribosylated muraymycin analogues **13** and **15** alongside the non-aminoribosylated reference compounds **14** and **16**. The design of the aminoribosylated target structures, particularly of **13**, has been inspired by an available X-ray co-crystal structure of the bacterial target protein MraY in complex with the naturally occurring inhibitor muraymycin D2 **9**. Constrasting the expectations, analogue **13** (with attachment of the aminoribosyl-linker unit to the 6′-amino group) was not active as an MraY inhibitor, but 6′-substituted non-aminoribosylated reference compound **14** was. The finding that uracil*-N-*3-derivatised analogues **15** and **16** were inactive as MraY inhibitors was in good agreement with predictions derived from the aforementioned co-crystal structure, but somewhat in contrast to the reported bioactivity of previously described muraymycin analogue **12**. The reason for this will be investigated in the context of future work, when we will synthesise and study muraymycin analogues **11** and **12** again to elucidate their interaction with MraY.

Taken together, the results for **13** and **14** suggest that the concept to derivatise the 6′-amino group of the muraymycin scaffold should be explored further in future work. In particular, it needs to be investigated if the introduction of more bulky substituents (such as an aminoribose unit) at this position is generally precluded, or if an approriate choice of linker length and architecture might furnish bioactive analogues. This will lead to valuable further SAR insights for muraymycins and will support the development of optimised analogues of this promising class of antibacterials. Work along this line is on the way in our laboratories.

## Figures and Tables

**Table 1:**
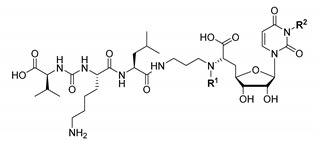
In vitro inhibitory activities of muraymycin analogues **13–16** against MraY from *S. aureus*.

compound	R^1^	R^2^	IC_50_ [µM] ^1^
**13**	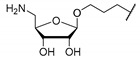	H	>100
**14**	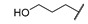	H	17 ± 9
αβ-**15**	H	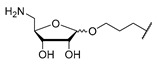	>100
**16**	H	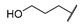	>100
**17**	H	H	2.5 ± 0.6

^1^ A crude membrane preparation of MraY from *S. aureus*, heterologously overexpressed in *E. coli*, was used. All measurements were carried out in triplicates. Data for **14** and **17** are mean value ± standard deviation.

## Supplementary Materials

The following are available online at http://www.mdpi.com/1420-3049/23/12/3085/s1, Figure S1, copies of NMR spectra.
