# Supplementary material for: Aminoribosylated Analogues of Muraymycin Nucleoside Antibiotics
Source: Molecules. 2018 Nov 26;23(12):3085. doi: 10.3390/molecules23123085 (PMC6320880; doi:10.3390/molecules23123085)
Supplement: Supplementary file 1 [file molecules-23-03085-s001.pdf]

Supplementary Materials

Aminoribosylated Analogues of Muraymycin Nucleoside Antibiotics

Daniel Wiegmann[1], Stefan Koppermann[1] and Christian Ducho[1,*]

[1] *Saarland University, Department of Pharmacy, Pharmaceutical and Medicinal Chemistry, Campus C2 3, 66 123 Saarbrücken, Germany*

*Corresponding author *E-mail: christian.ducho@uni-saarland.de*

Table of contents

^{1}H and ^{13}C NMR spectra of synthesised compounds.. S2

^{1}H and ^{13}C NMR spectra of synthesised compounds

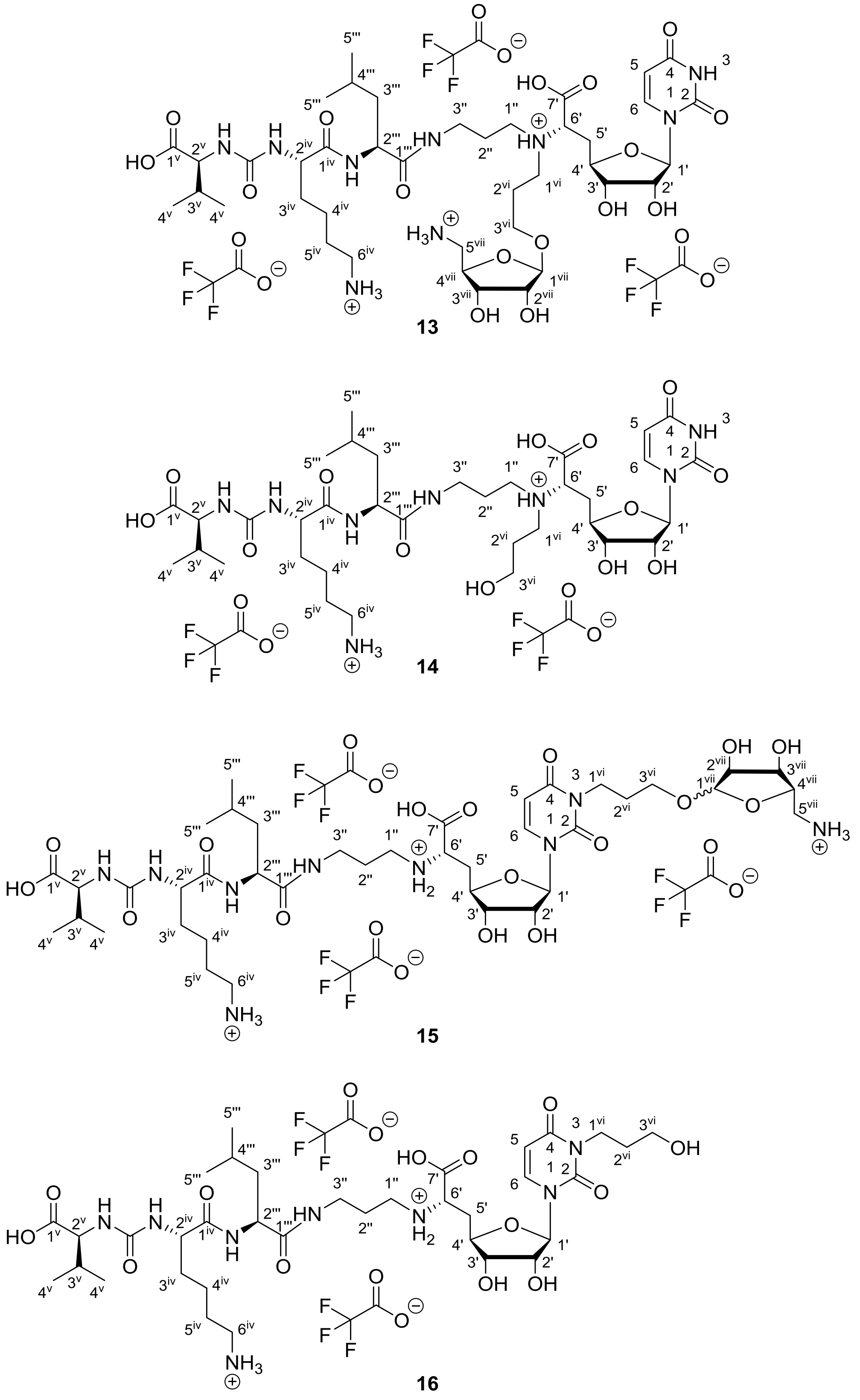

Figure S1. Numbering of atoms of muraymycin target structures **13-16** for the assignment of NMR signals.

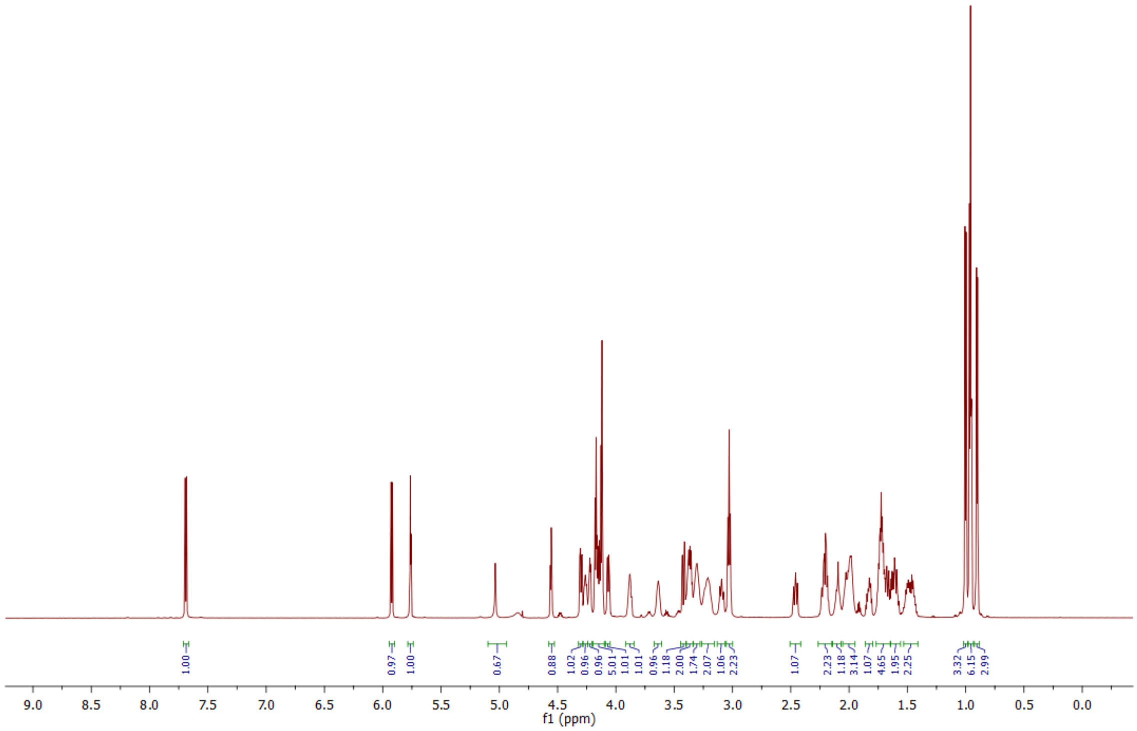

^{1}H NMR spectrum of **13** (500 MHz, D_2O).

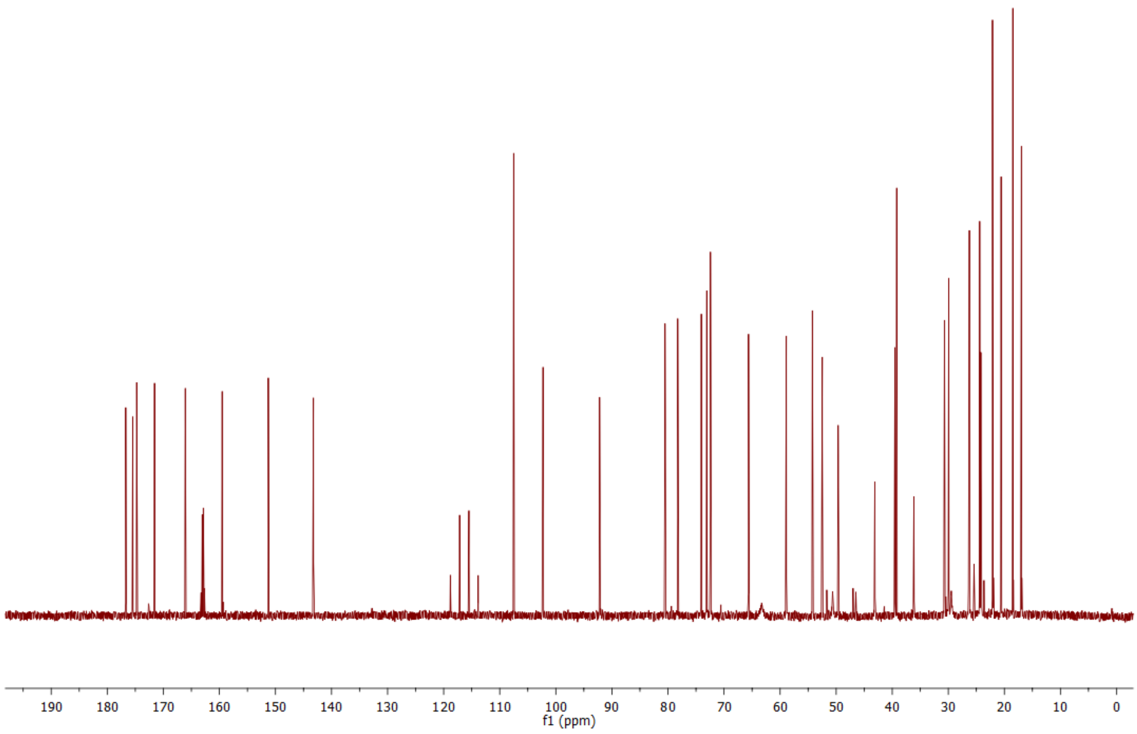

^{13}C NMR spectrum of **13** (126 MHz, D_2O).

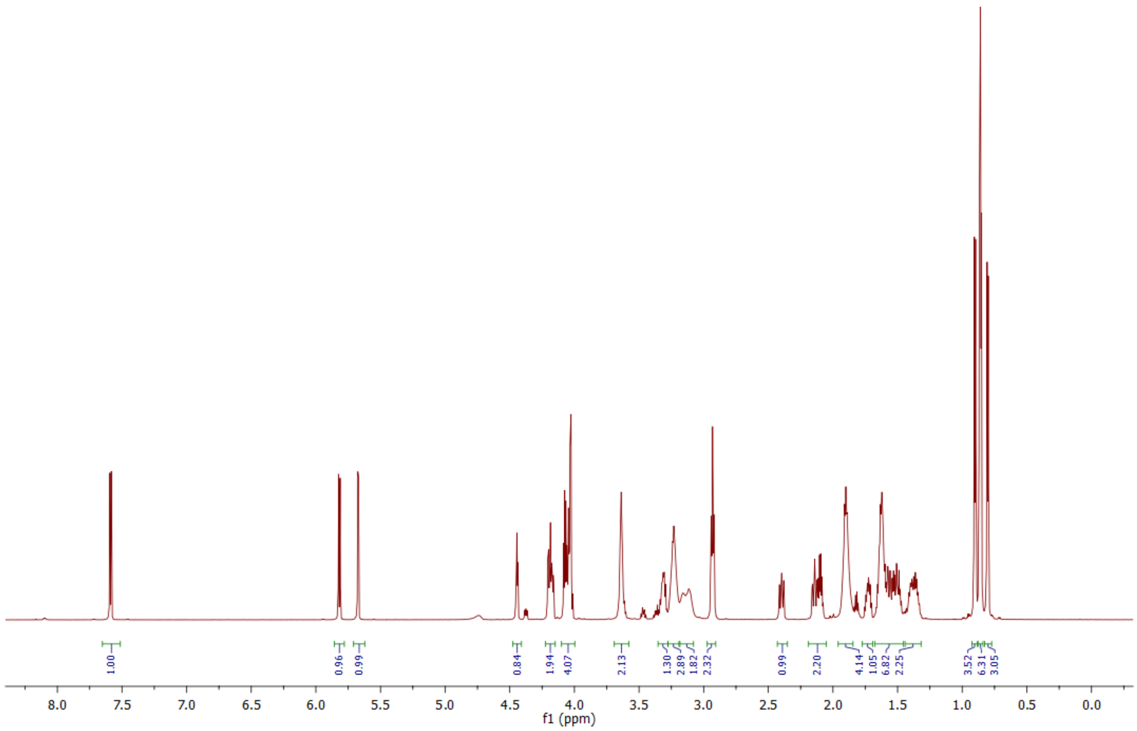

^{1}H NMR spectrum of **14** (500 MHz, D_2O).

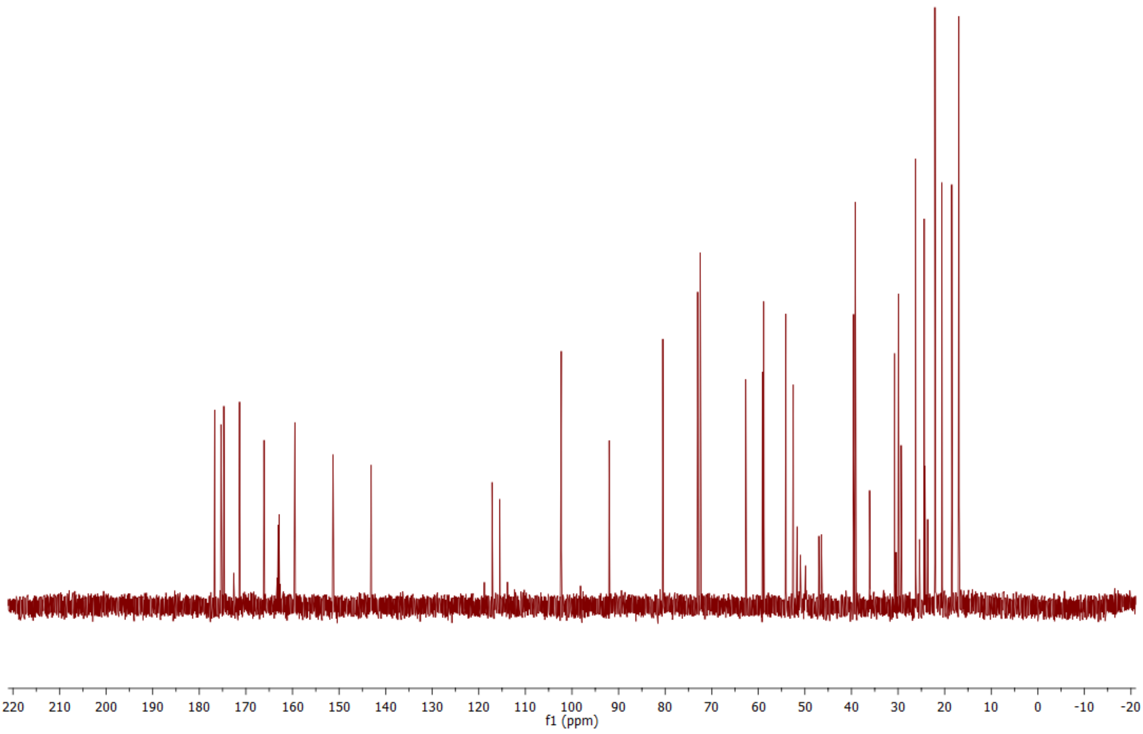

^{13}C NMR spectrum of **14** (126 MHz, D_2O).

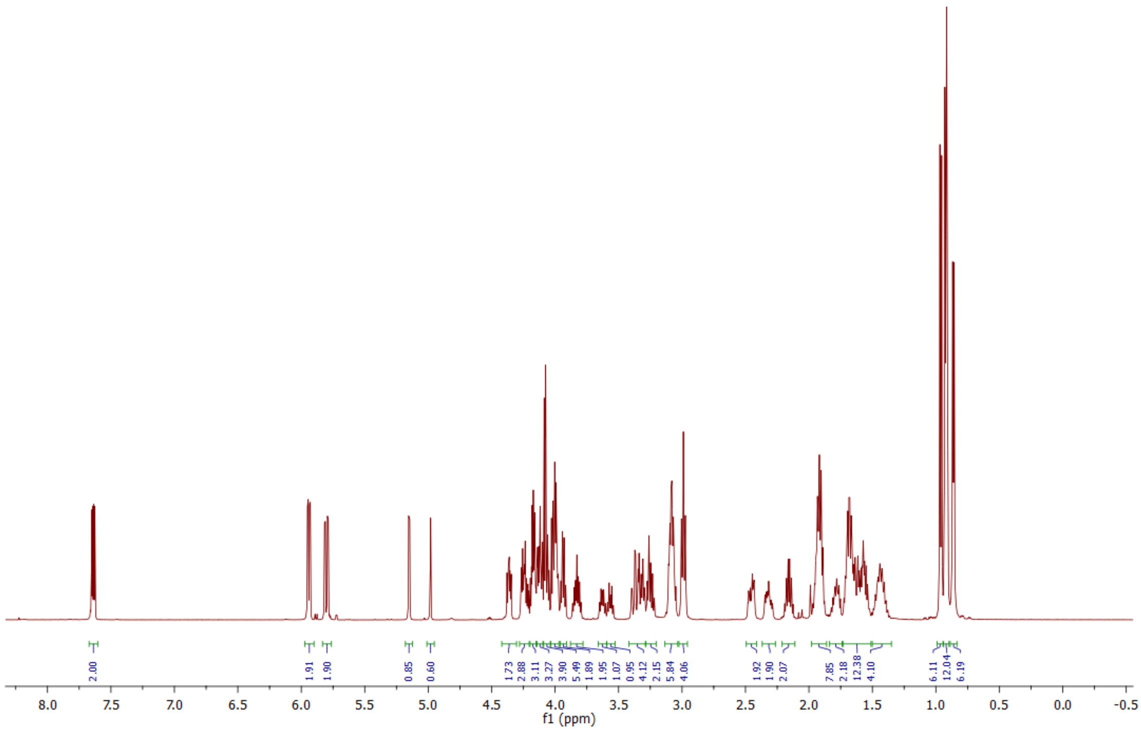

^{1}H NMR spectrum of αβ-**15** (500 MHz, D_2O).

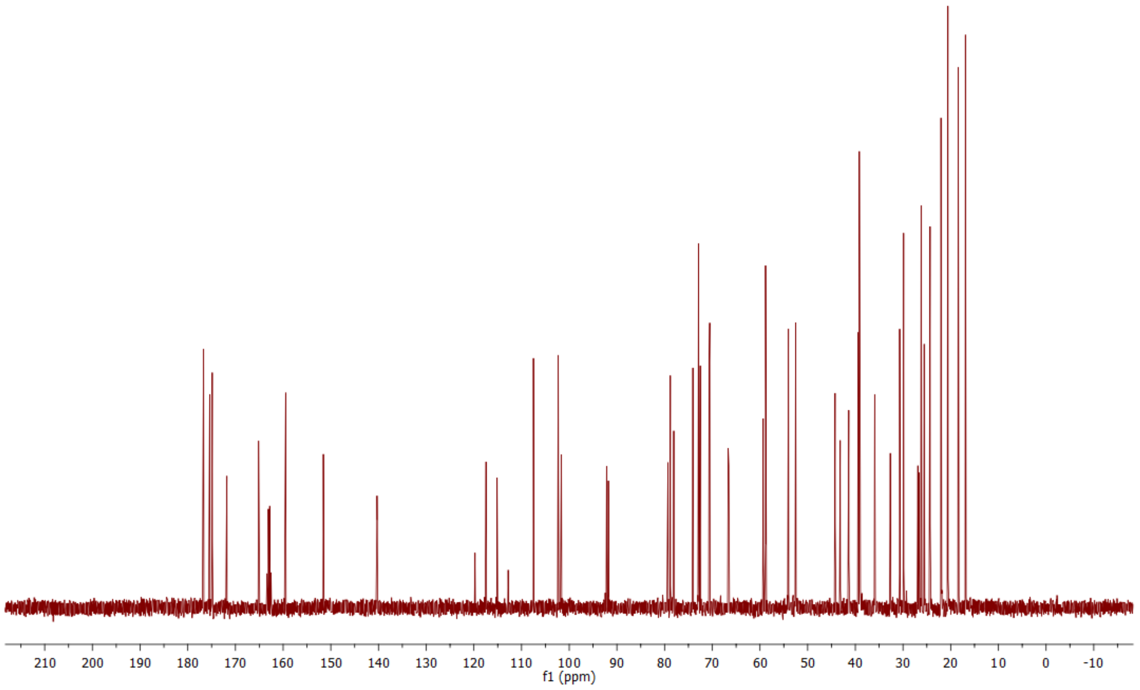

^{13}C NMR spectrum of αβ-**15** (126 MHz, D_2O).

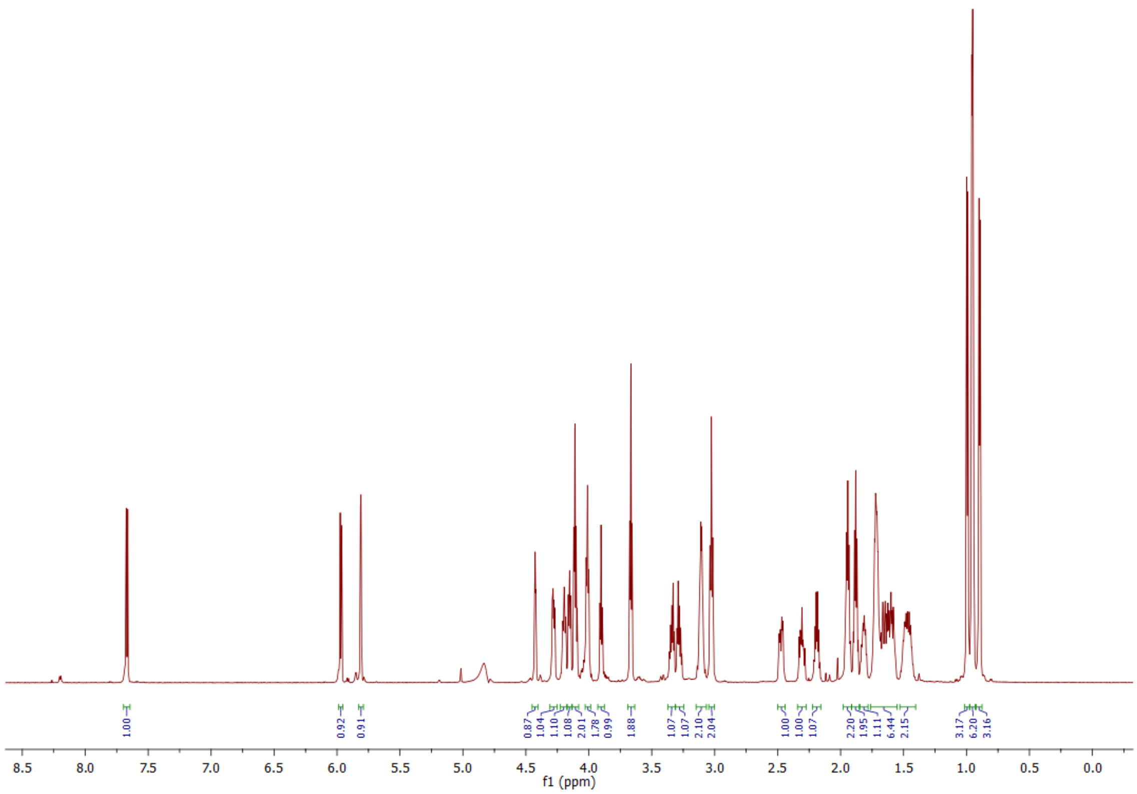

^{1}H NMR spectrum of **16** (500 MHz, D_2O).

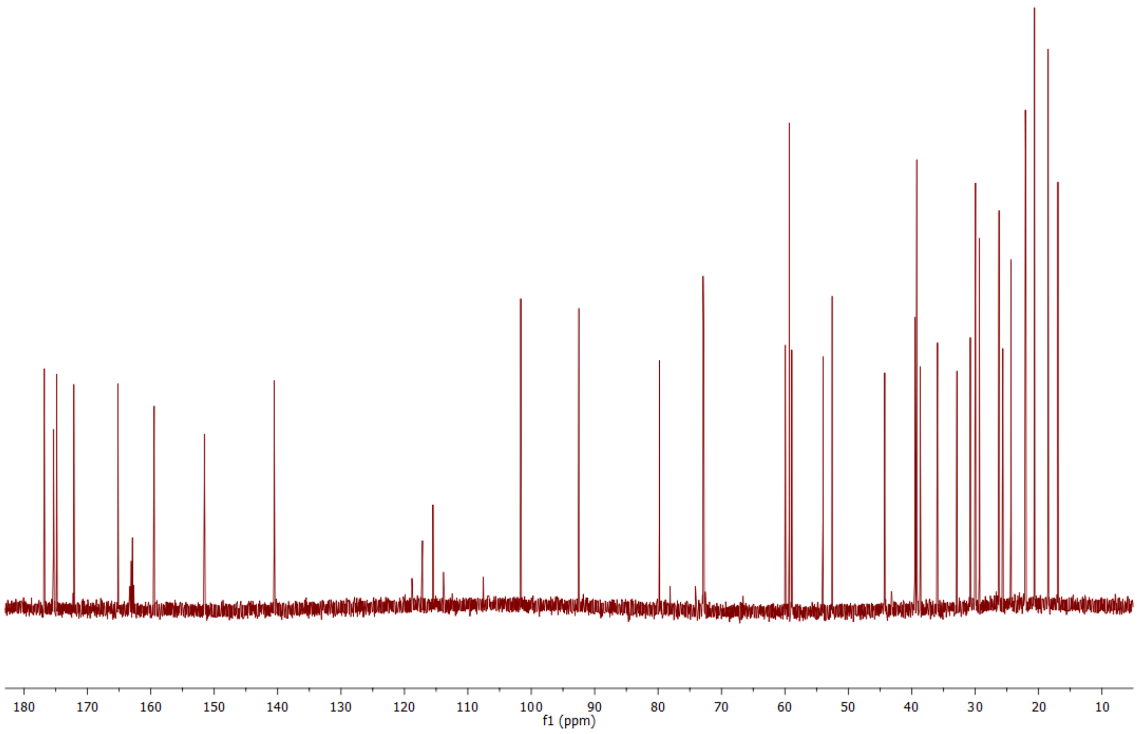

^{13}C NMR spectrum of **16** (126 MHz, D_2O).

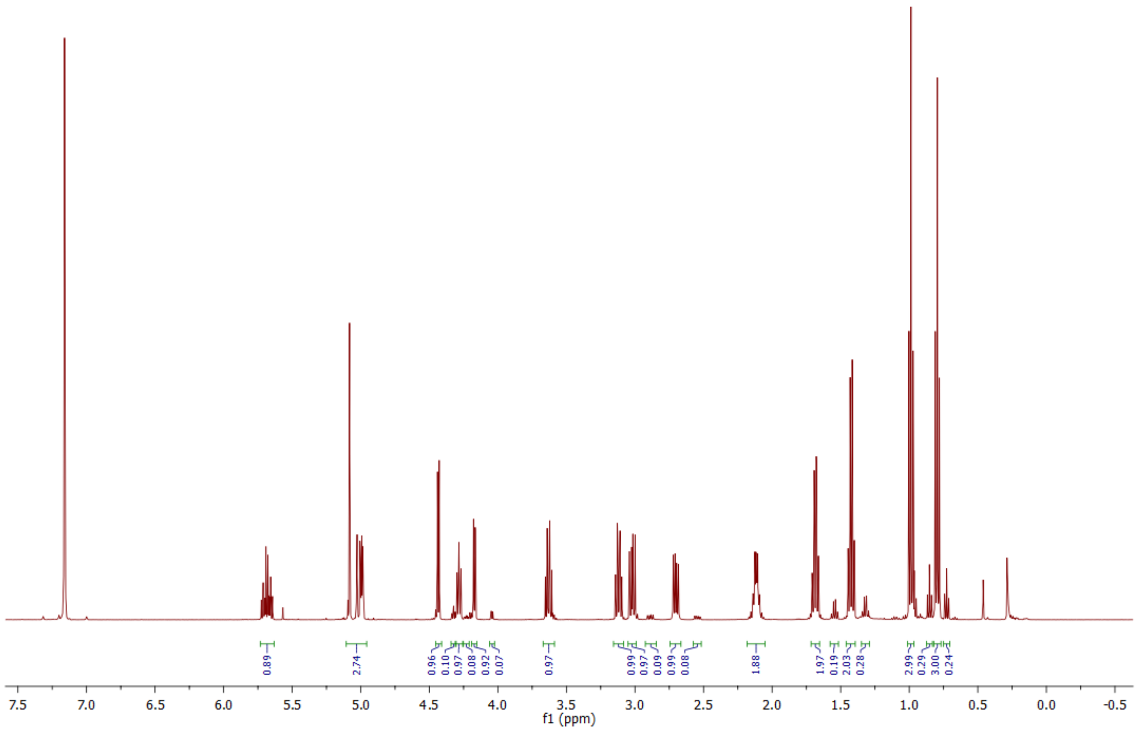

^{1}H NMR spectrum of **20** (500 MHz, C_6D_6).

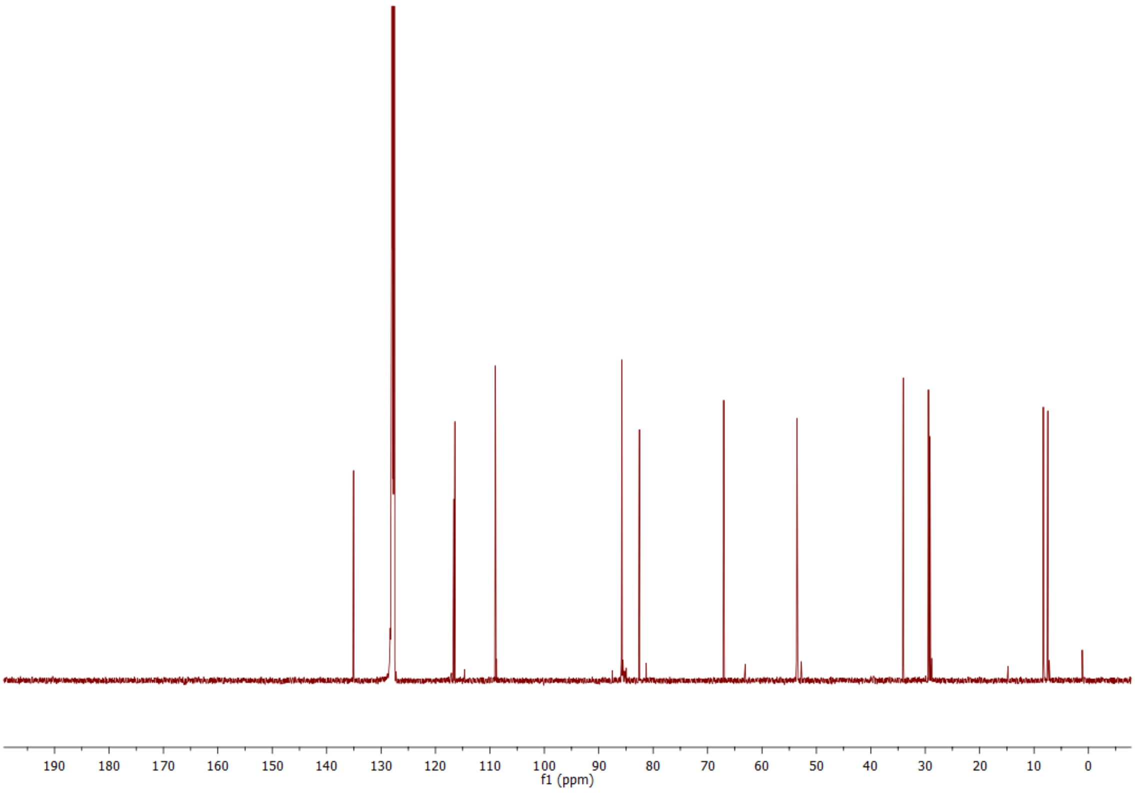

^{13}C NMR spectrum of **20** (126 MHz, C_6D_6).

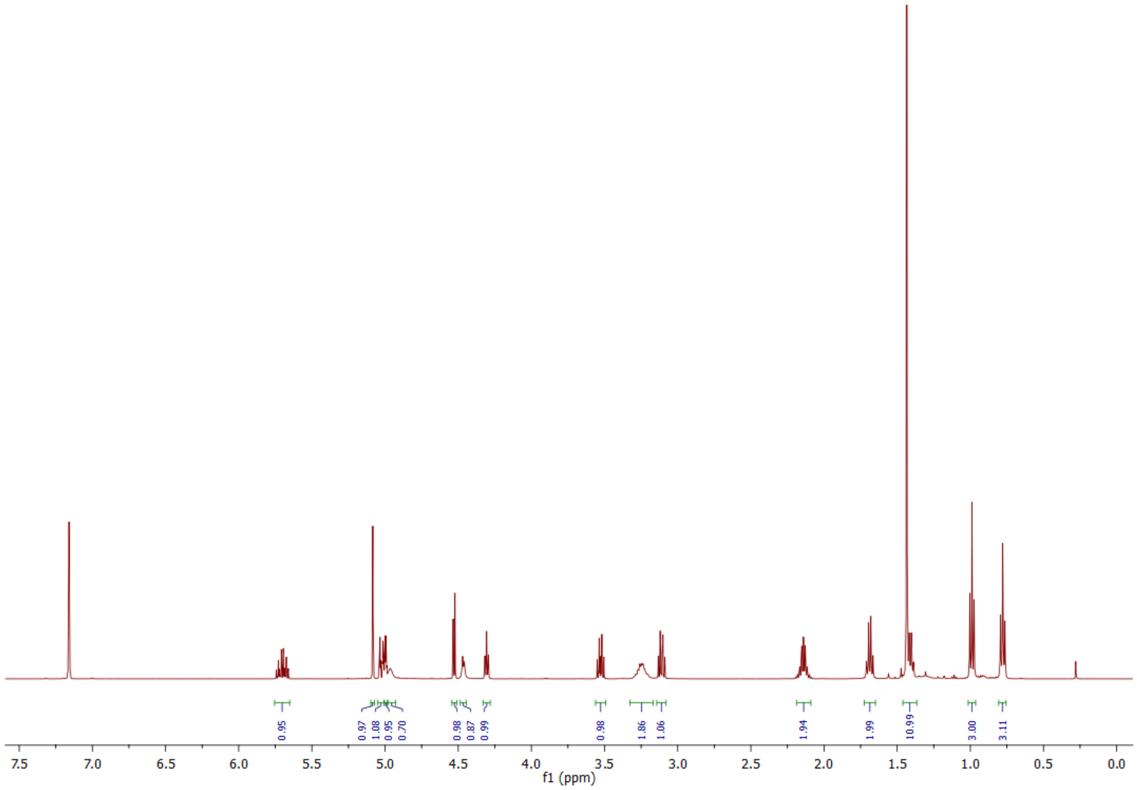

^{1}H NMR spectrum of **21** (500 MHz, C_6D_6).

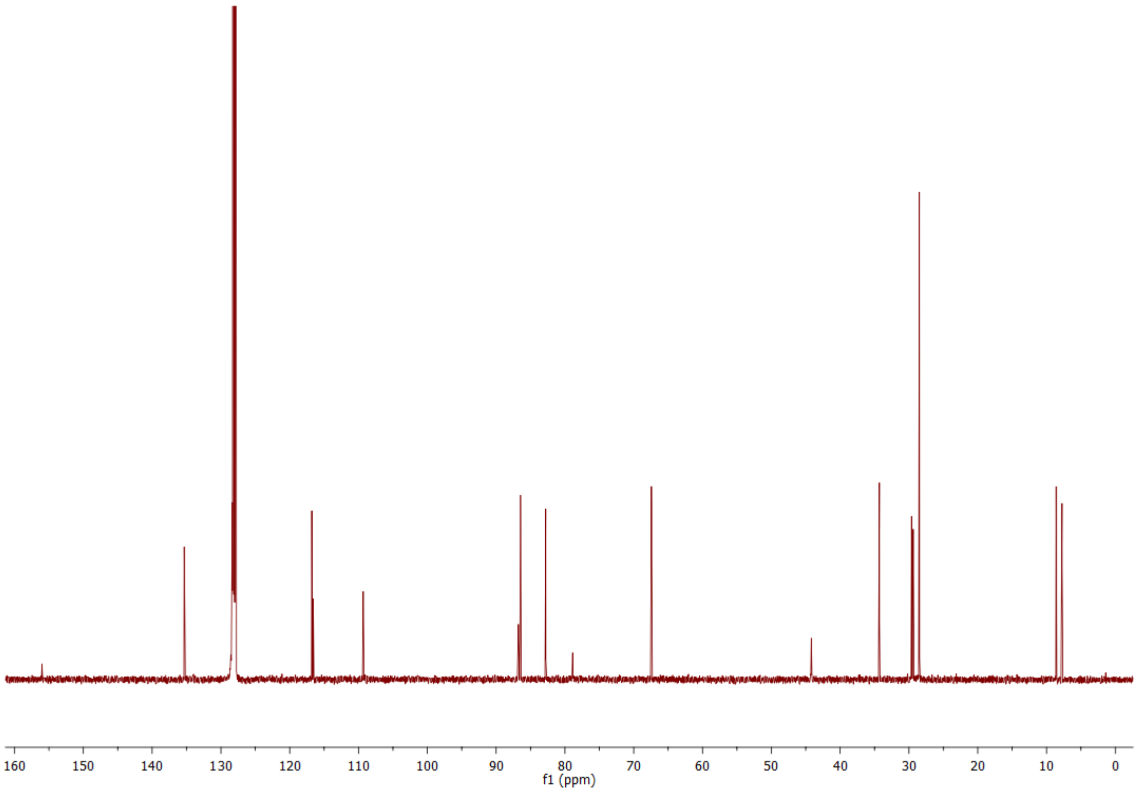

^{13}C NMR spectrum of **21** (126 MHz, C_6D_6).

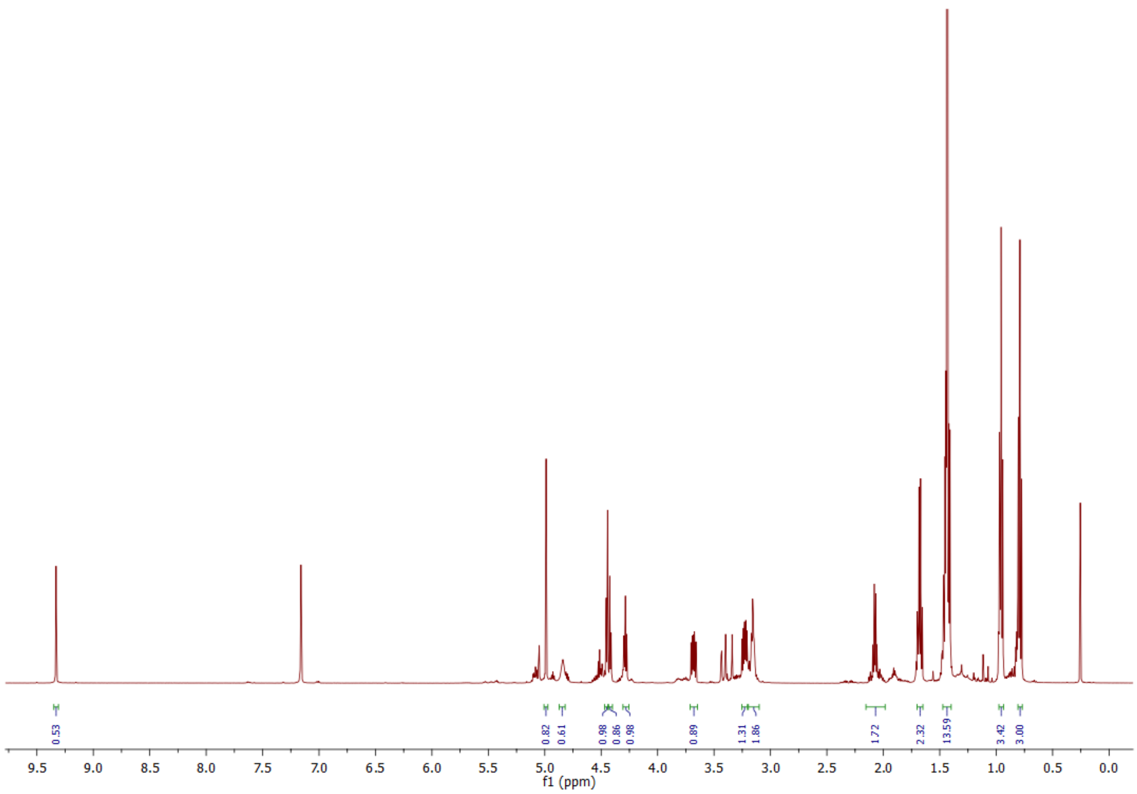

^{1}H NMR spectrum of **22** (500 MHz, C_6D_6, 70 °C).

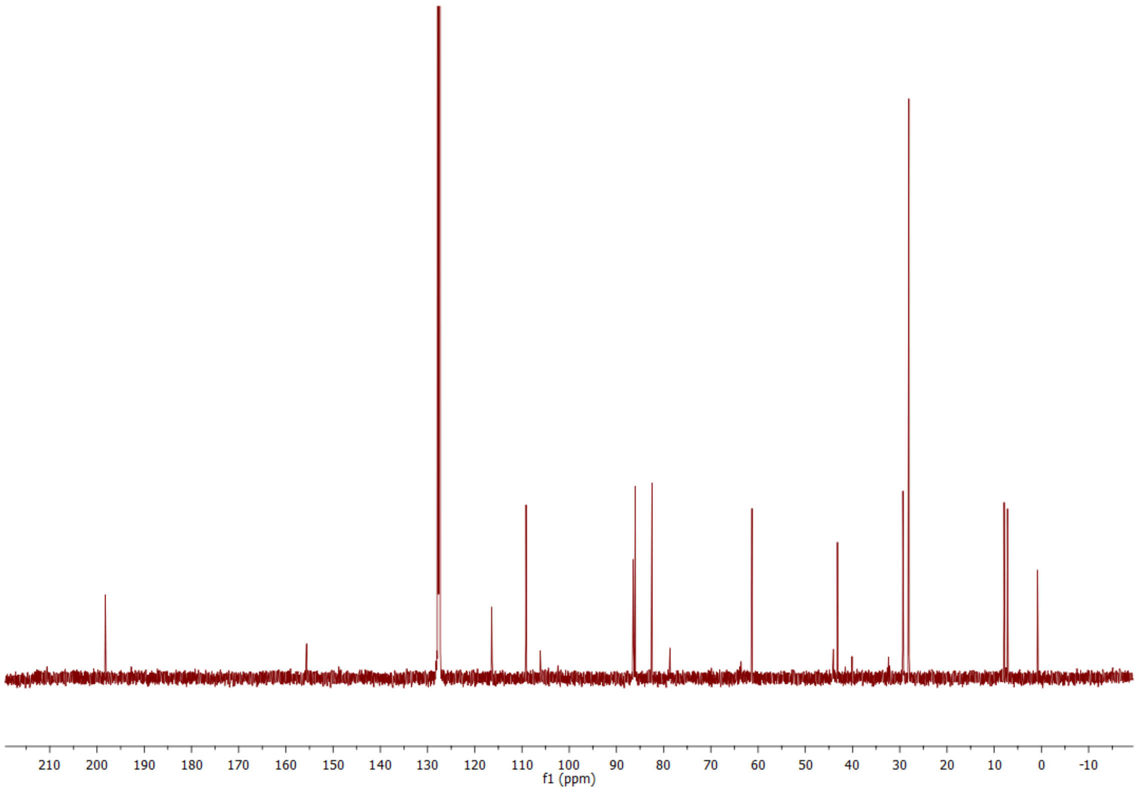

^{13}C NMR spectrum of **22** (126 MHz, C_6D_6, 70 °C).

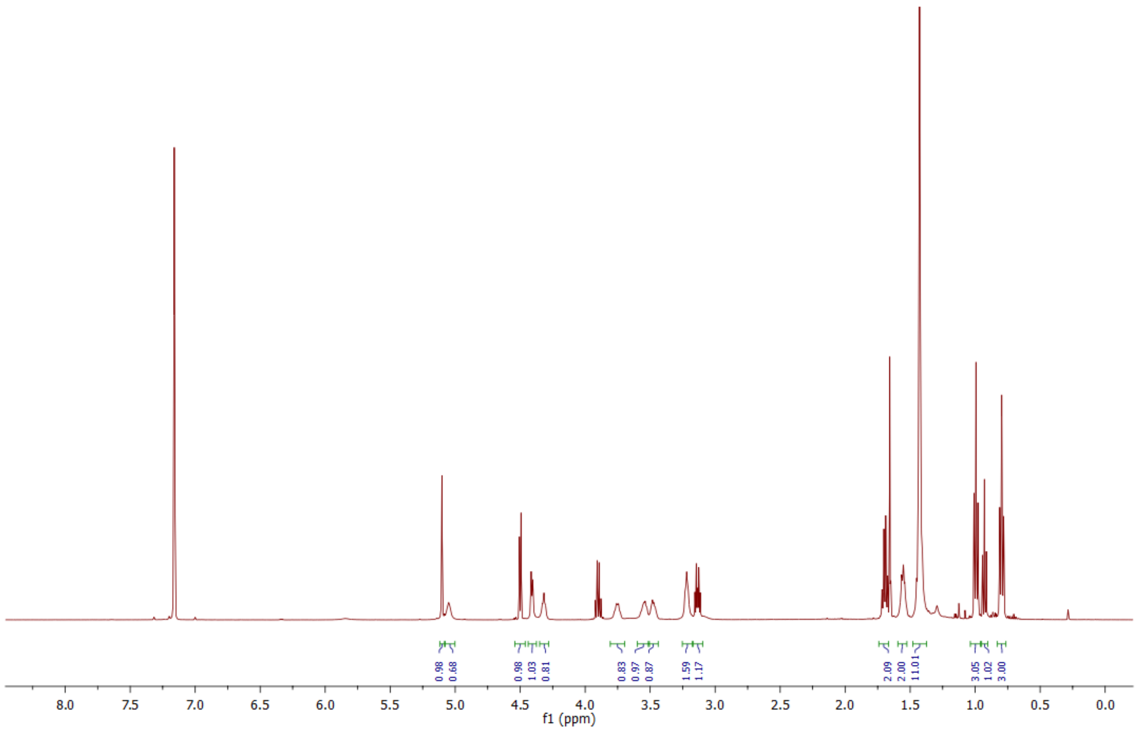

^{1}H NMR spectrum of **23** (500 MHz, C_6D_6).

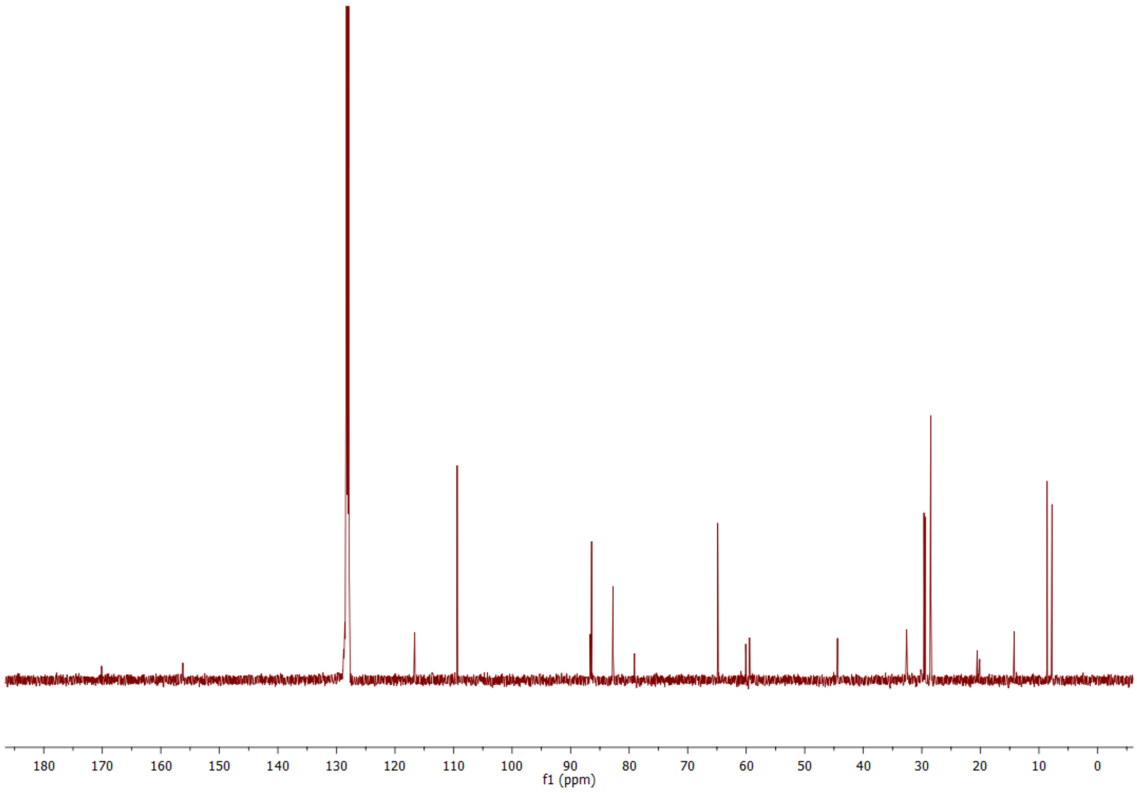

^{13}C NMR spectrum of **23** (126 MHz, C_6D_6).

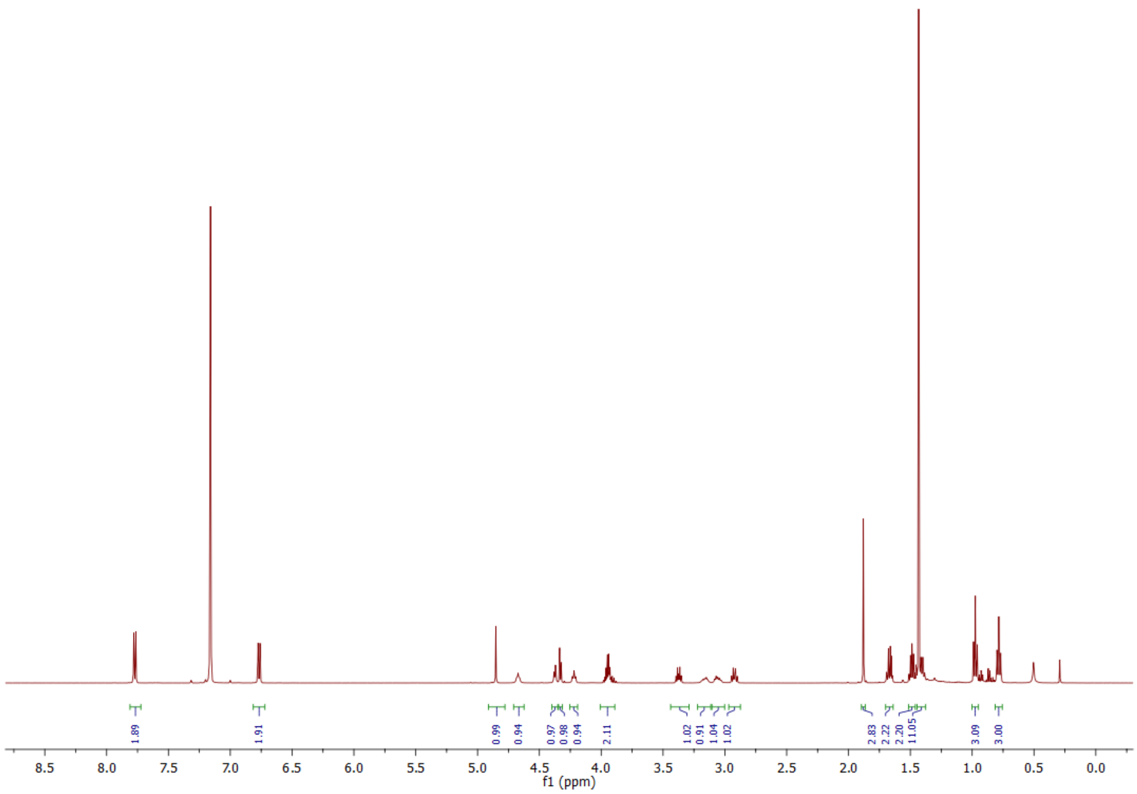

^{1}H NMR spectrum of **24** (500 MHz, C_6D_6).

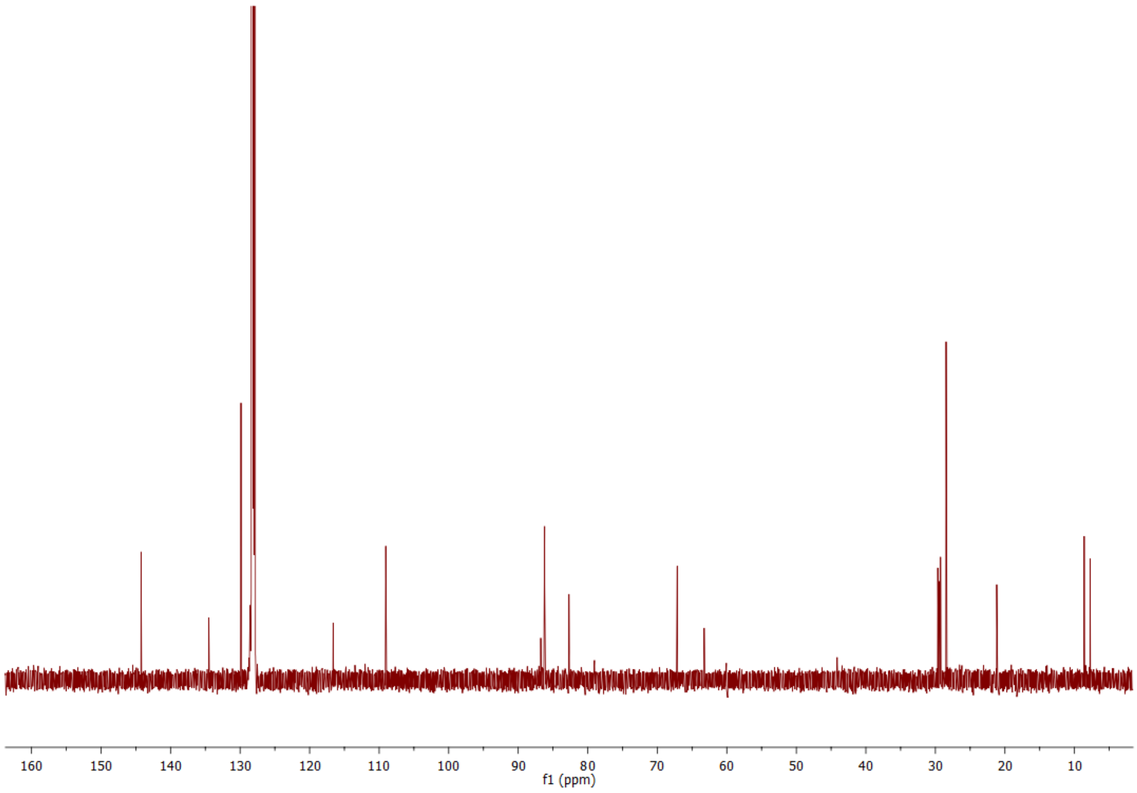

^{13}C NMR spectrum of **24** (126 MHz, C_6D_6).

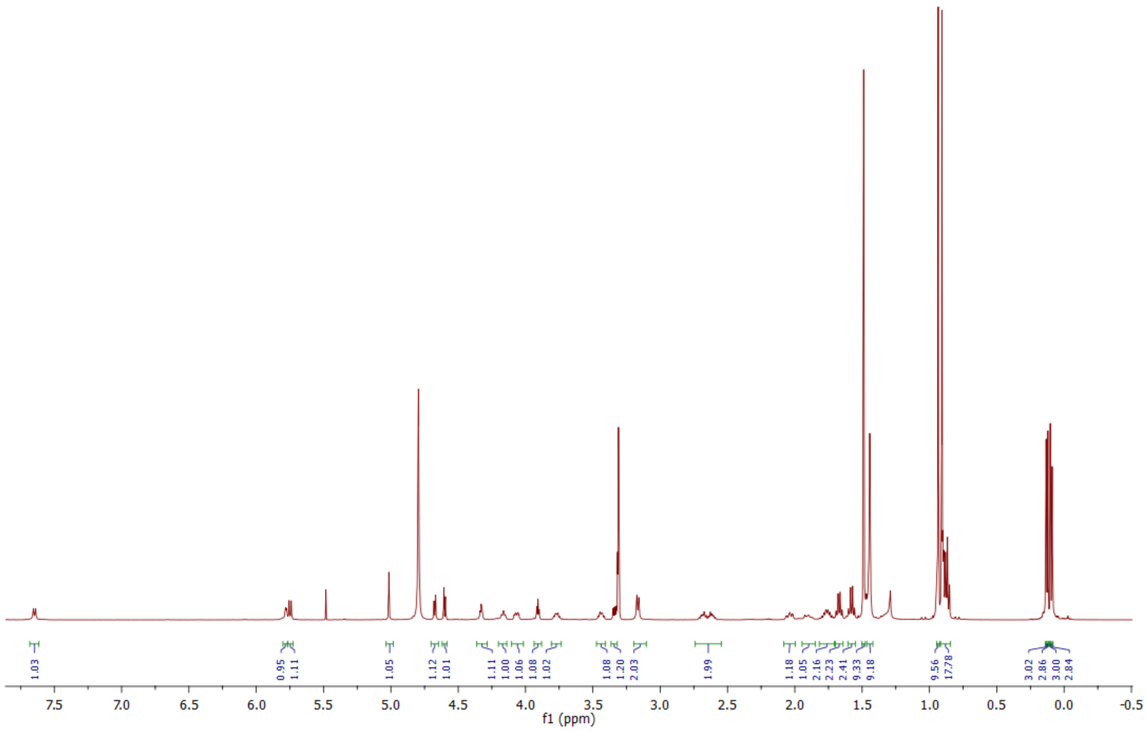

^{1}H NMR spectrum of **26** (500 MHz, CD_3OD).

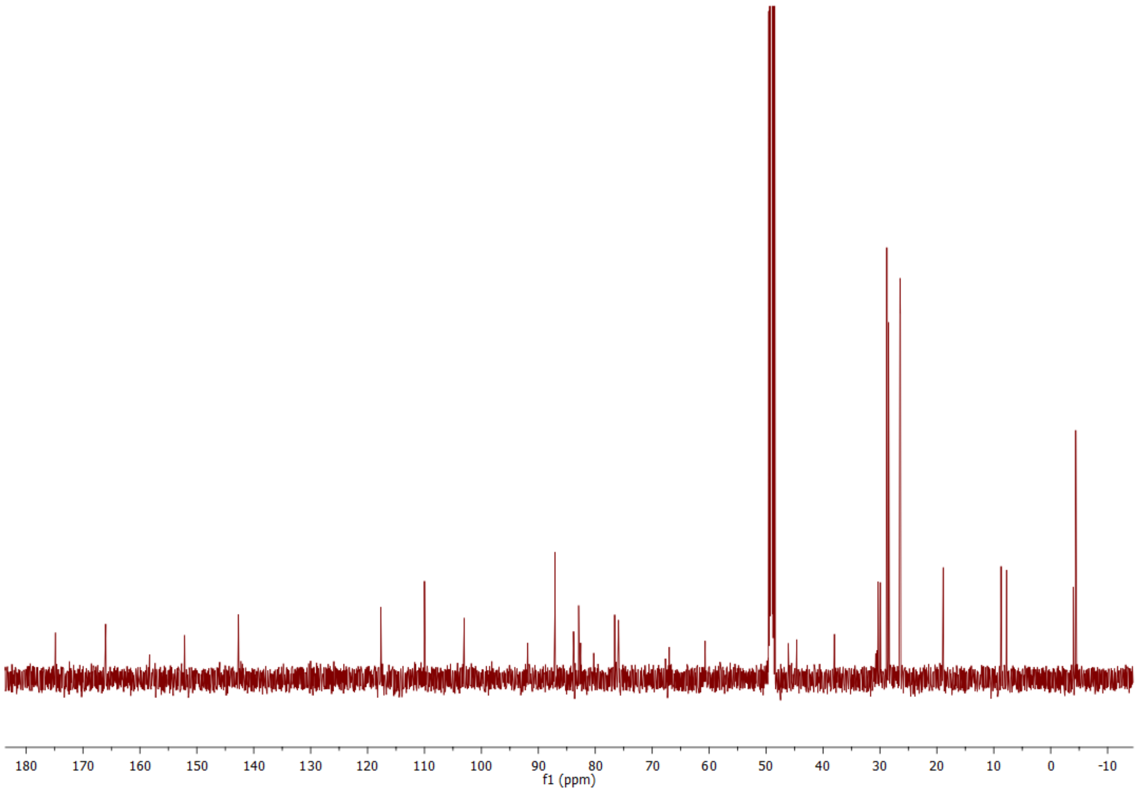

^{13}C NMR spectrum of **26** (126 MHz, CD_3OD).

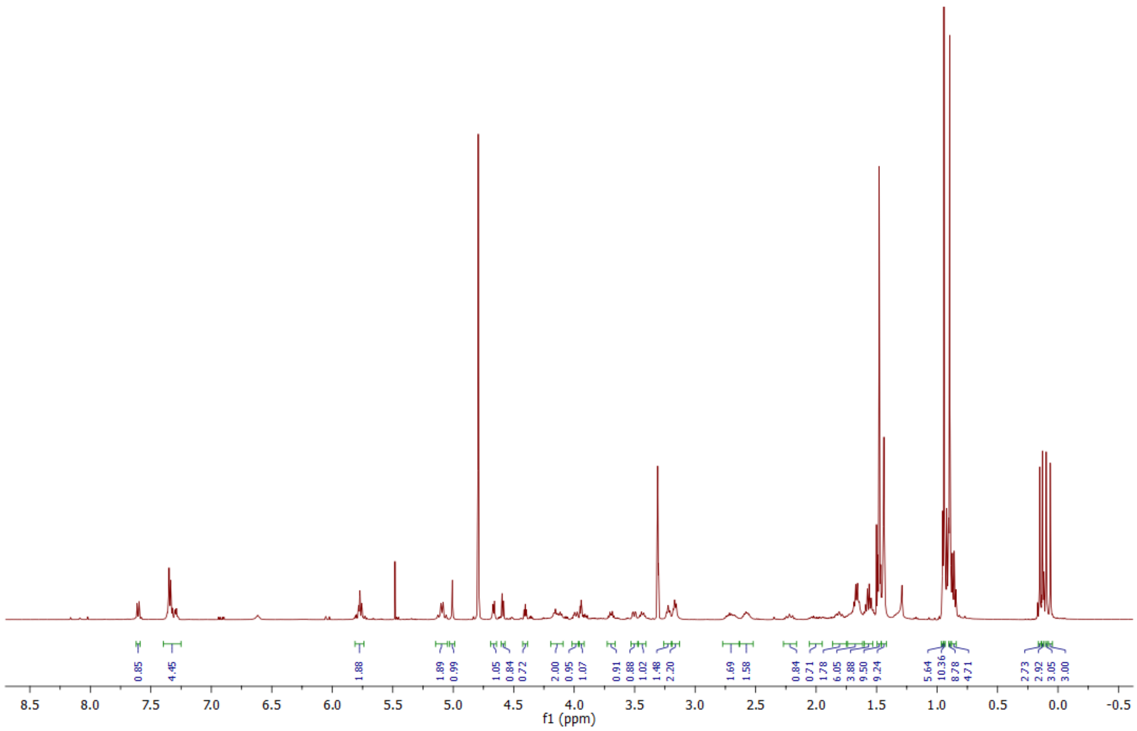

^{1}H NMR spectrum of **28** (500 MHz, CD_3OD).

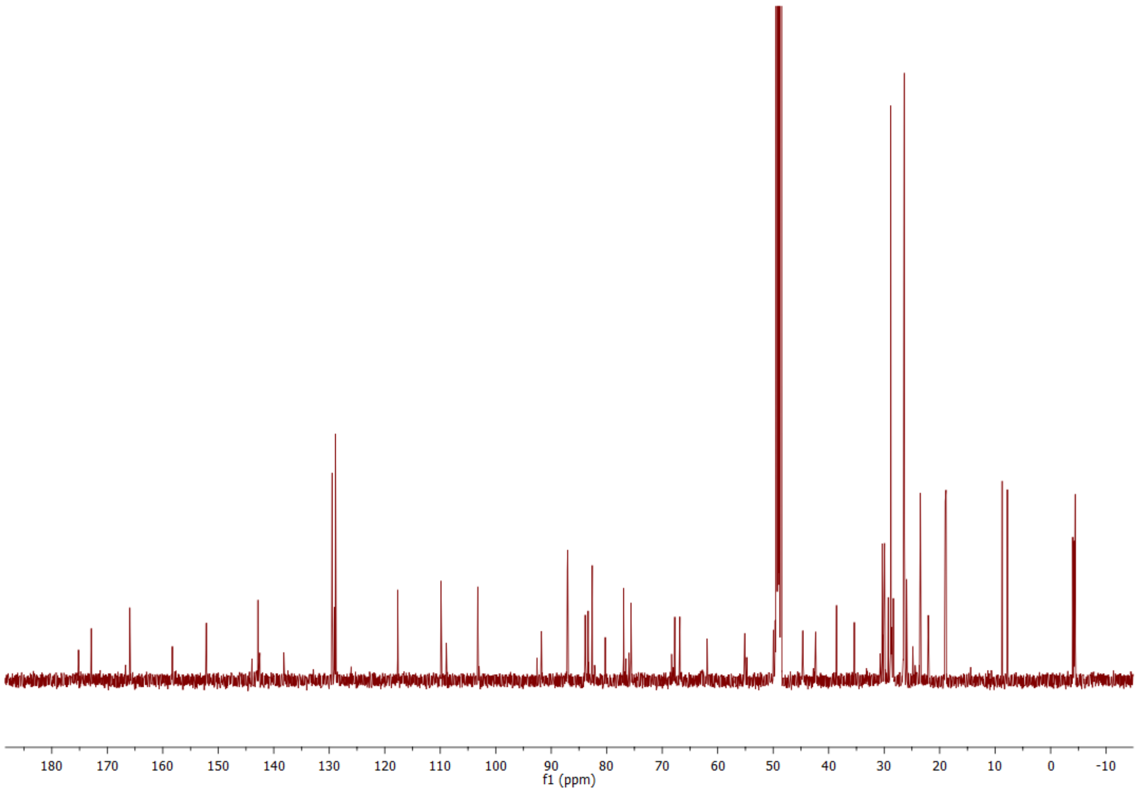

^{13}C NMR spectrum of **28** (126 MHz, CD_3OD).

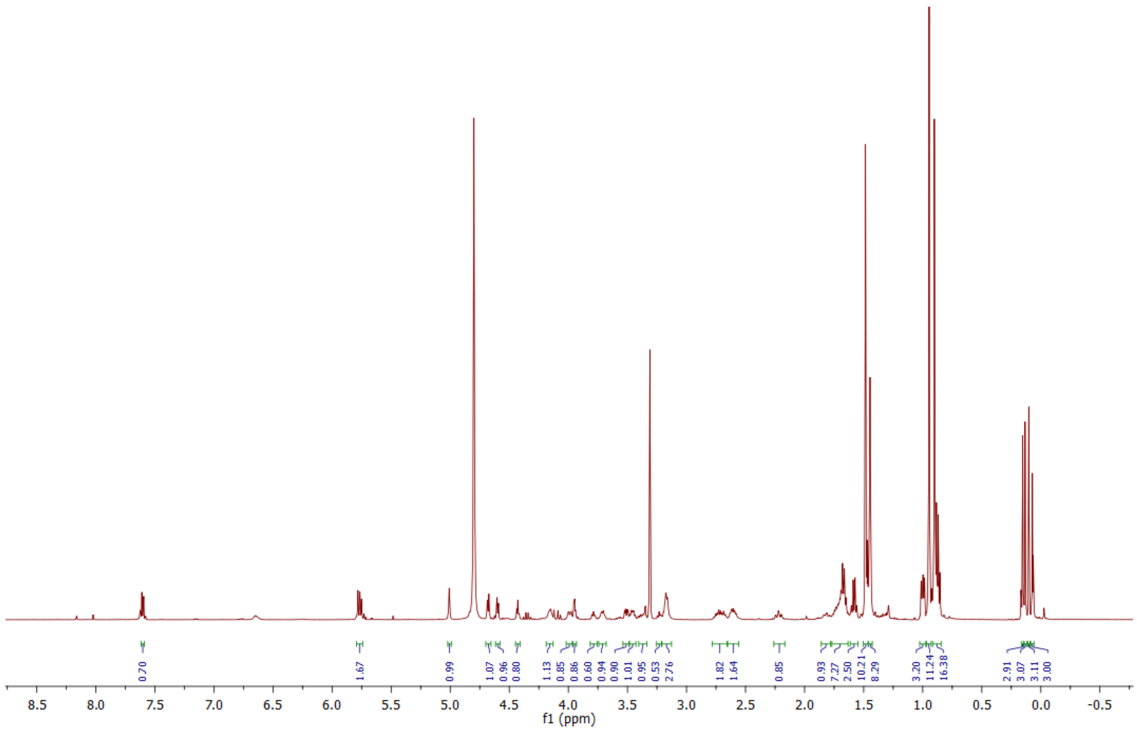

^{1}H NMR spectrum of **29** (500 MHz, CD_3OD).

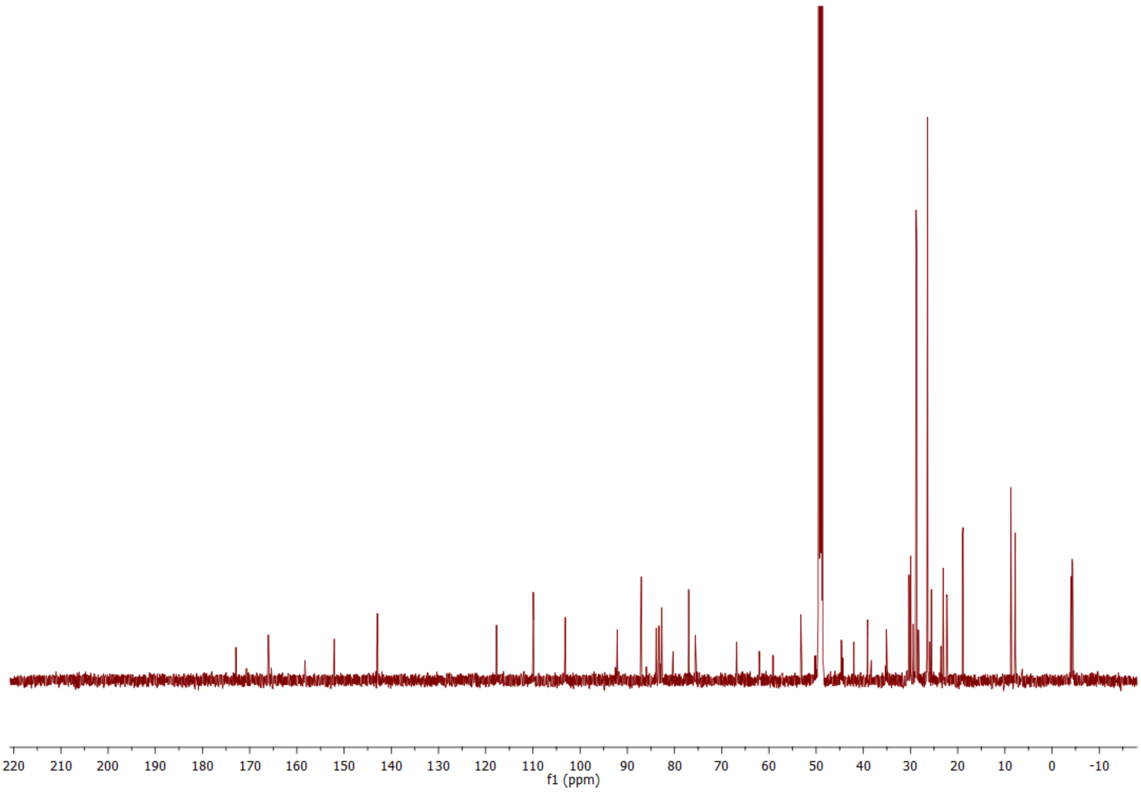

^{13}C NMR spectrum of **29** (126 MHz, CD_3OD).

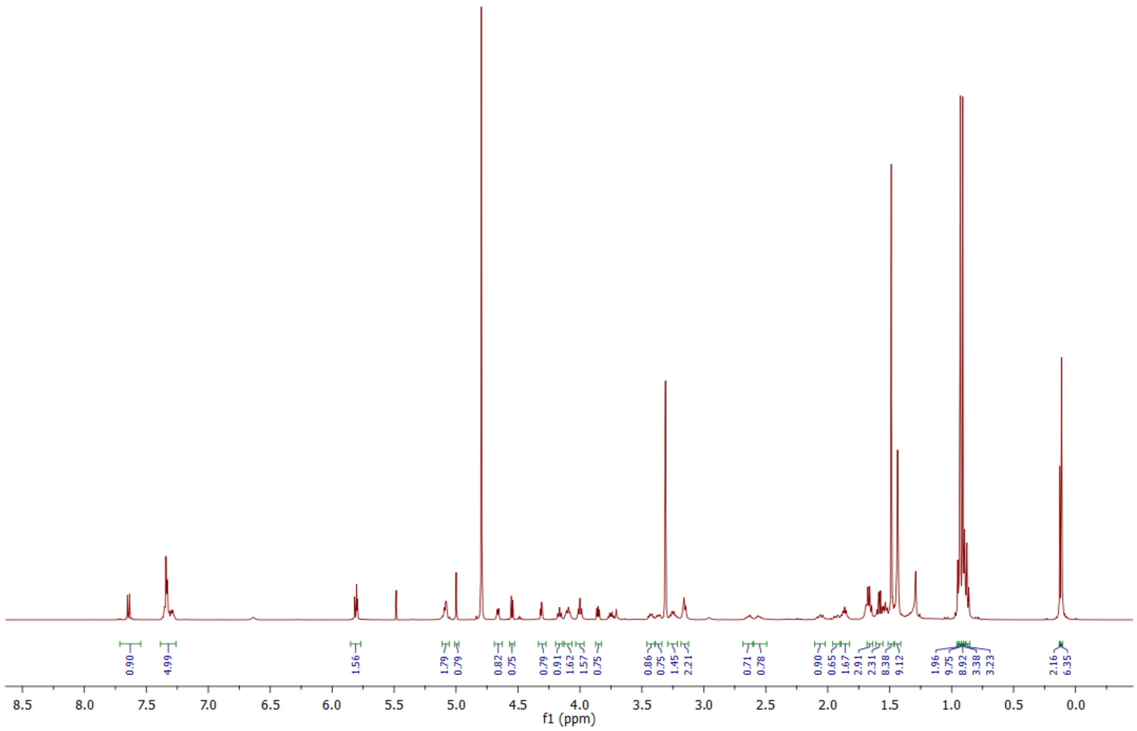

^{1}H NMR spectrum of **33** (500 MHz, CD_3OD).

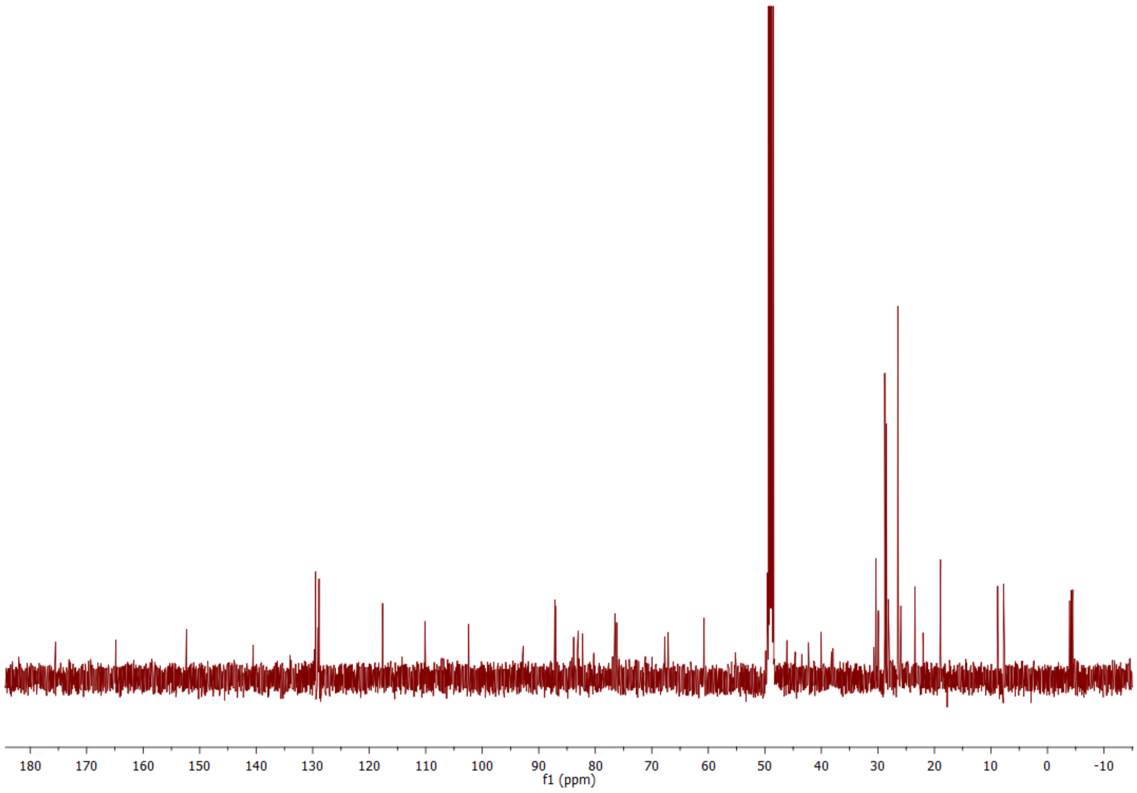

^{13}C NMR spectrum of **33** (126 MHz, CD_3OD).
